# Supplementary material for: HDAC3 Regulates the Transition to the Homeostatic Myelinating Schwann Cell State
Source: Cell Rep. 2018 Dec 4;25(10):2755–2765.e5. doi: 10.1016/j.celrep.2018.11.045 (PMC6293966; doi:10.1016/j.celrep.2018.11.045)
Supplement: Document S1. Figures S1–S6 and Tables S1 and S2 [file mmc1.pdf]

Cell Reports, Volume *25*

Supplemental Information

HDAC3 Regulates the Transition to the Homeostatic Myelinating Schwann Cell State

Laura H. Rosenberg, Anne-Laure Cattin, Xavier Fontana, Elizabeth Harford-Wright, Jemima J. Burden, Ian J. White, Jacob G. Smith, Ilaria Napoli, Victor Quereda, Cristina Policarpi, Jamie Freeman, Robin Ketteler, Antonella Riccio, and Alison C. Lloyd

Figure S1

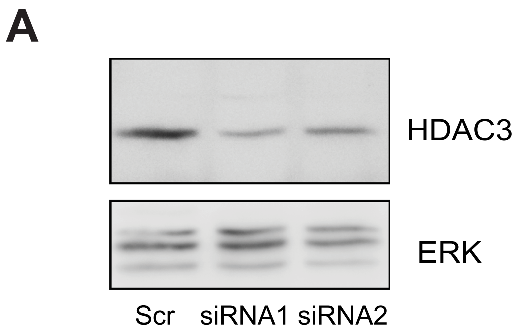
A
HDAC3
ERK
Scr
siRNA1
siRNA2

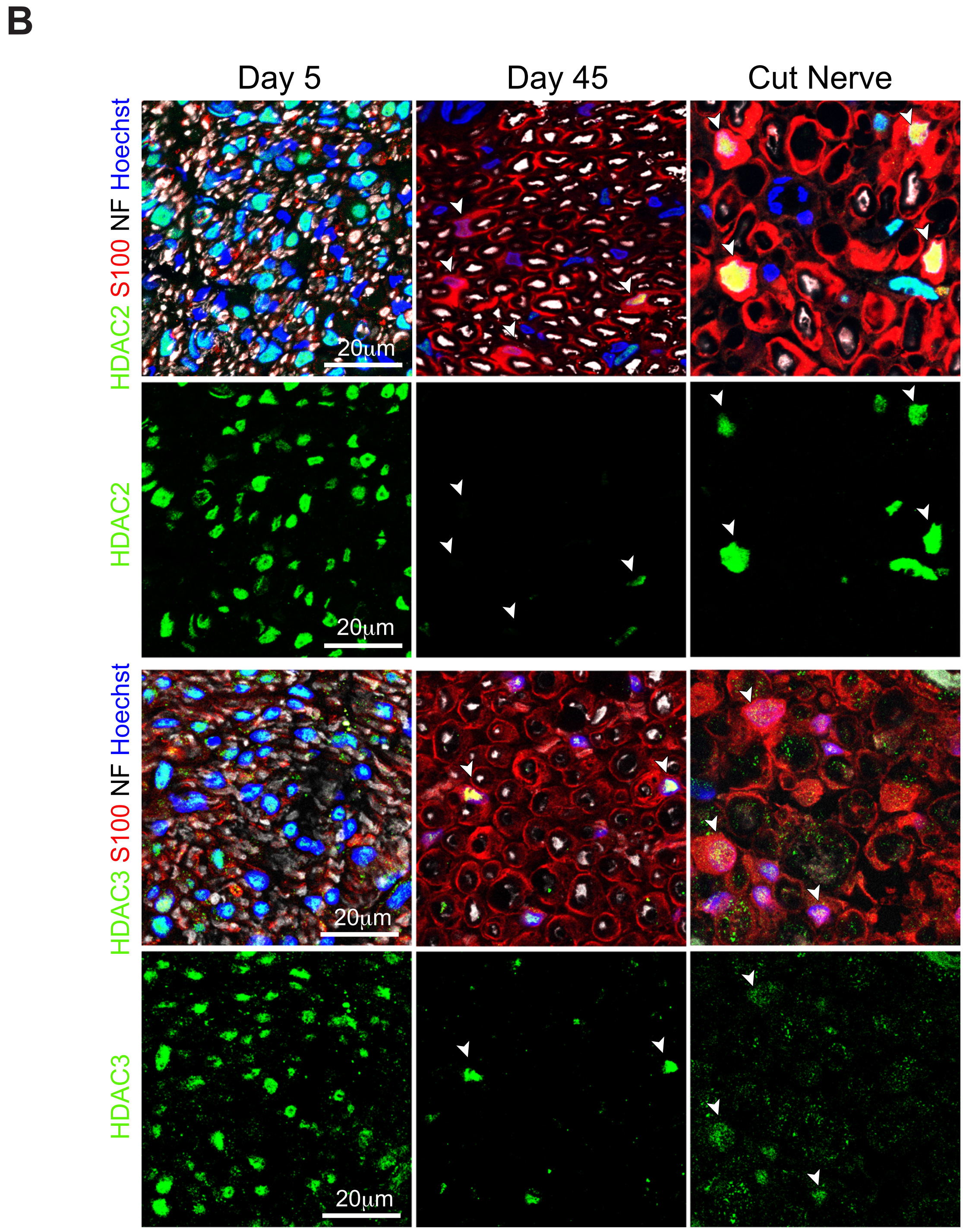
B
Day 5
Day 45
Cut Nerve
HDAC2 S100 NF Hoechst
HDAC2
HDAC3 S100 NF Hoechst
HDAC3
20µm
20µm
20µm
20µm

Supplementary Figure 1. HDAC3 regulates myelin gene transcription and is expressed in adult myelinating Schwann cells, related to Figure 1. A) Western blot analysis of total protein lysates from siRNA-treated Schwann cells showing the efficiency of HDAC3 knockdown with two independent oligos compared to scrambled control at 48 hr. Total ERK levels were used as a loading control. B) Representative confocal images of transverse sections of rat sciatic nerves at postnatal Day 5, Day 45, and Day 45 animals, 72 hours following nerve transection stained for S100 (red), neurofilament (white) and HDAC2 (top panel) or HDAC3 (lower panel) (green), as indicated. Nuclei were counterstained with Hoechst (blue). Note that similarly to mice (Figure 1D), HDAC2 expression is low in adult mSCs whereas it is reinduced upon injury (arrowheads). Conversely HDAC3 expression is maintained in adult mSCs (arrowheads) whereas it decreases upon injury (arrowheads).

Figure S2

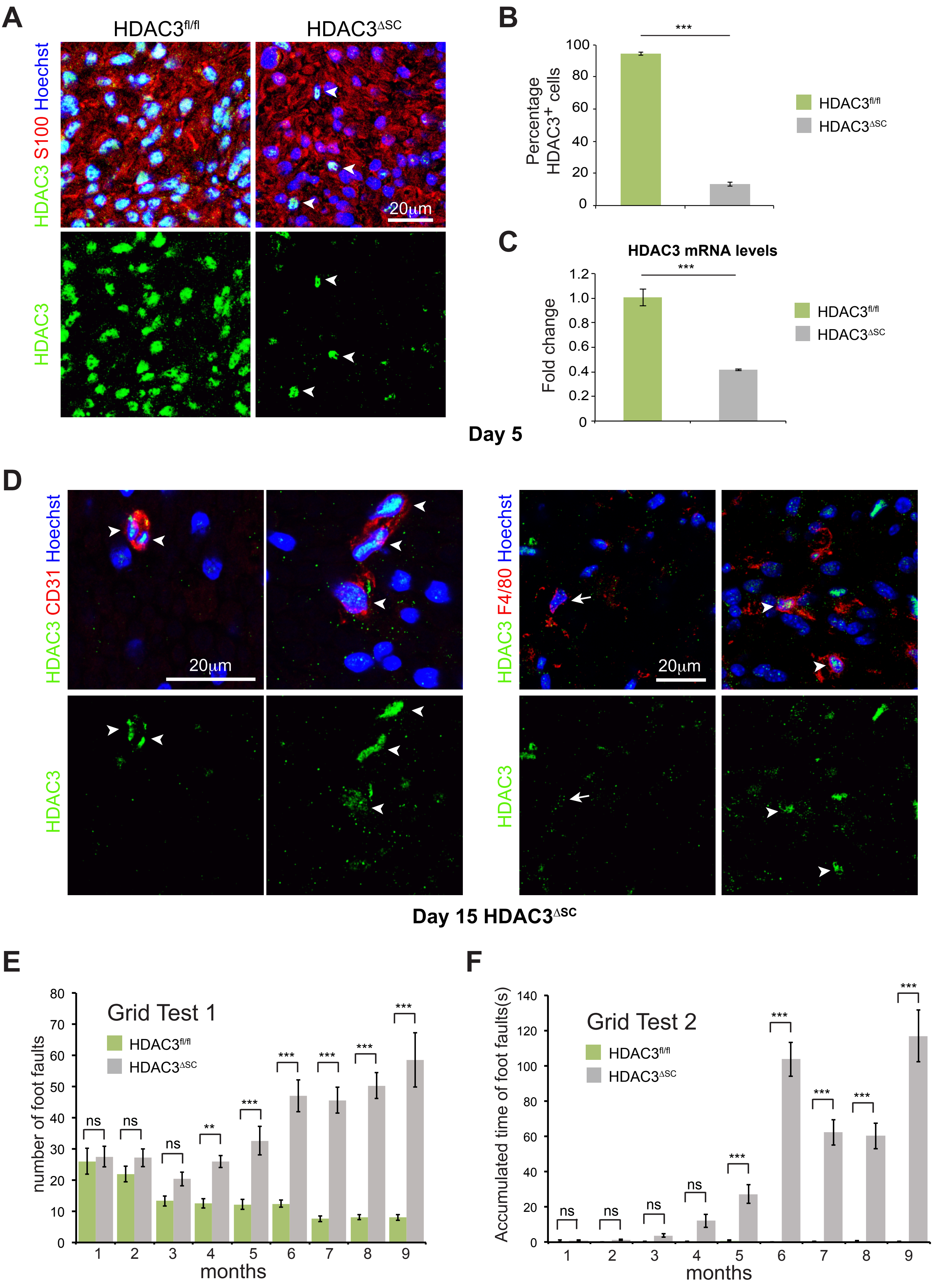

A
HDAC3fl/fl
HDAC3ΔSC
HDAC3 S100 Hoechst
HDAC3
20μm
B
Percentage HDAC3+ cells
100
80
60
40
20
0
***
HDAC3fl/fl
HDAC3ΔSC
C
HDAC3 mRNA levels
Fold change
1.2
1.0
0.8
0.6
0.4
0.2
0
***
HDAC3fl/fl
HDAC3ΔSC
Day 5
D
HDAC3 CD31 Hoechst
HDAC3
20μm
HDAC3 F4/80 Hoechst
HDAC3
20μm
Day 15 HDAC3ΔSC
E
Grid Test 1
HDAC3fl/fl
HDAC3ΔSC
number of foot faults
80
70
60
50
40
30
20
10
0
ns
ns
ns
**
***
***
***
***
***
1
2
3
4
5
6
7
8
9
months
F
Grid Test 2
HDAC3fl/fl
HDAC3ΔSC
Accumulated time of foot faults(s)
140
120
100
80
60
40
20
0
ns
ns
ns
ns
***
***
***
***
***
1
2
3
4
5
6
7
8
9
months

Supplementary Figure 2. Loss of HDAC3 in Schwann cells results in progressive adult neuropathy, related to Figure 2. A) Immunofluorescence of representative transverse sciatic nerve sections from control (HDAC3$^{fl/fl}$) mice or mutant mice (HDAC3$^{\Delta SC}$) at postnatal Day 5 showing efficient loss of nuclear HDAC3 staining (green) in S100-labelled Schwann cells (red). Nuclei were counterstained with Hoechst (blue). Arrowheads point to other cell types that still express HDAC3 in mutant mice. B) Graph shows quantification of (A), which indicates a significant decrease of cells expressing HDAC3 in mutant compared to control mice (n=4, mean±SEM). (C) RT-qPCR analysis of HDAC3 mRNA levels in control (HDAC3$^{fl/fl}$) and mutant mice (HDAC3$^{\Delta SC}$) at postnatal Day 5 (n=3, mean±SEM). (D) Representative confocal images of transverse sections of sciatic nerve from mutant mice (HDAC3$^{\Delta SC}$) at postnatal Day 15 showing HDAC3 staining (green) in endothelial cells labelled with CD31 and indicated with arrowheads (left panel) and in macrophages labelled with F4/80 (right panel) (red). Note only some macrophages are positive for HDAC3 with arrowheads marking HDAC+ macrophages and an arrow indicating a HDAC3- macrophage. Nuclei were counterstained with Hoechst (blue). E) and (F) Grid behavioural test showing (E) average number and (F) accumulated time of foot faults in seconds of control (HDAC3$^{fl/fl}$) and mutant (HDAC3$^{\Delta SC}$) animals from 1-9 months after birth (n=4-19 animals/time point, mean±SEM). $^{**}p<0.01$, $^{***}p<0.001$.

Movie S1, related to Figure 2. Adult HDAC3$^{\Delta SC}$ mice display severe motor disabilities.

Video showing an example of a 9 month-old mutant HDAC3$^{\Delta SC}$ mouse showing symptoms consistent with an advanced neuropathy.

Figure S3

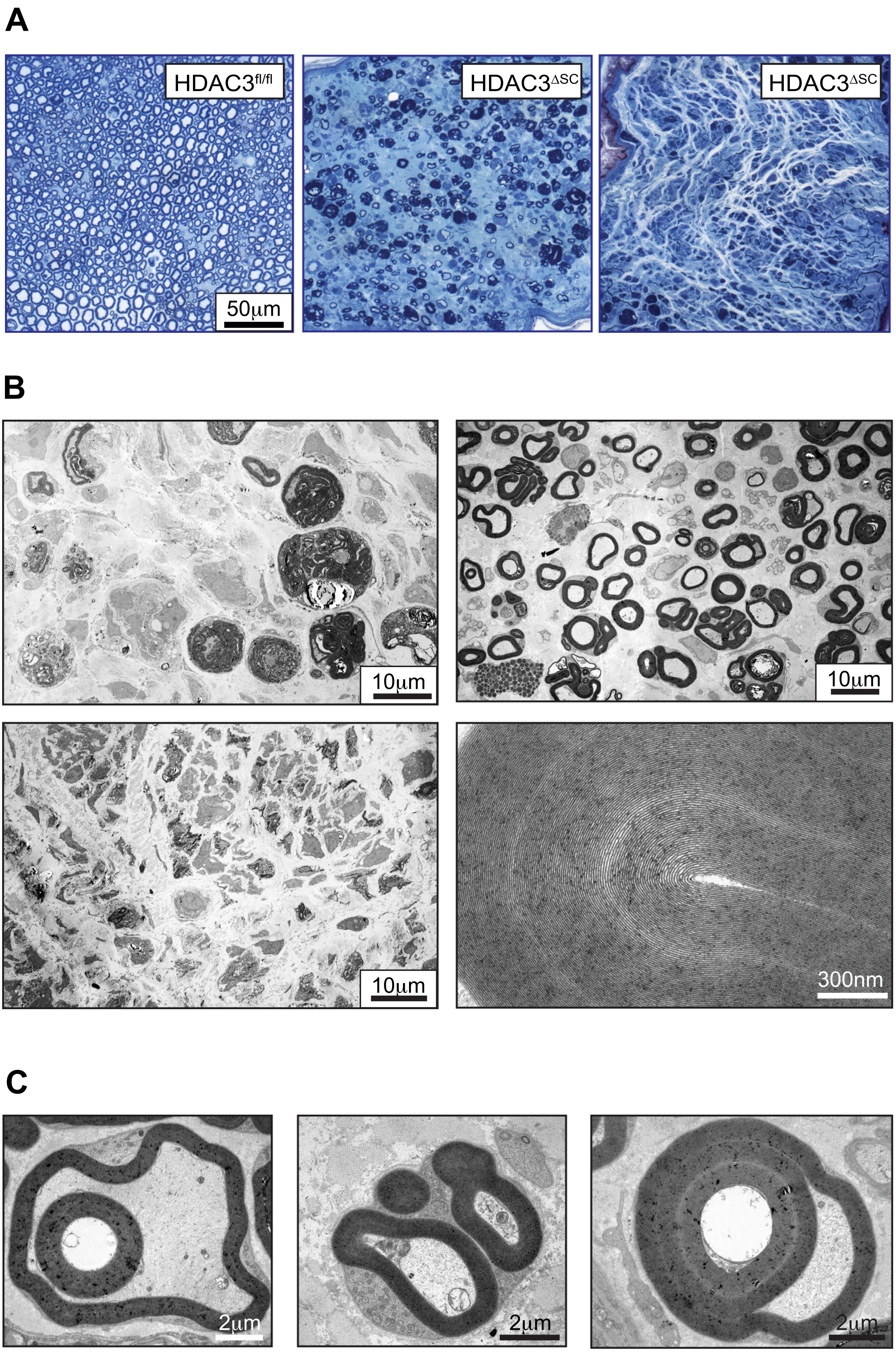
A
HDAC3$^{fl/fl}$
HDAC3$^{\Delta SC}$
HDAC3$^{\Delta SC}$
50μm
B
10μm
10μm
10μm
300nm
C
2μm
2μm
2μm

Supplementary Figure 3. HDAC3 loss in Schwann cells results in gross myelinating abnormalities, related to Figure 3. (A) Representative images of transverse sections of a sciatic nerve from 9 month-old control HDAC3$^{fl/fl}$ and mutant HDAC3$^{\Delta SC}$ mice stained with toluidine blue, when the mutant animals exhibit severe neuropathies. The left image from a mutant animal shows a decrease in myelinated fibre number and myelination abnormalities, the right image shows fibrotic tissue and predominantly naked axons in more extreme cases. (B) Representative EM images of transverse ultrathin sections showing a collection of gross abnormalities in SC-axonal units in mutant HDAC3$^{\Delta SC}$ mice. Left panels show examples of fibrotic tissue resulting from Schwann cell death and axonal degeneration. The top right panel illustrates abnormalities due to hypermyelination such as tomacula, outfoldings and infoldings as well as inflammatory cell recruitment. The bottom right panel shows normal structure and compaction of myelin in mutant HDAC3$^{\Delta SC}$ mice. (C) High magnification EM images showing myelin infoldings (left and right panel) and outfoldings (middle panel).

Figure S4

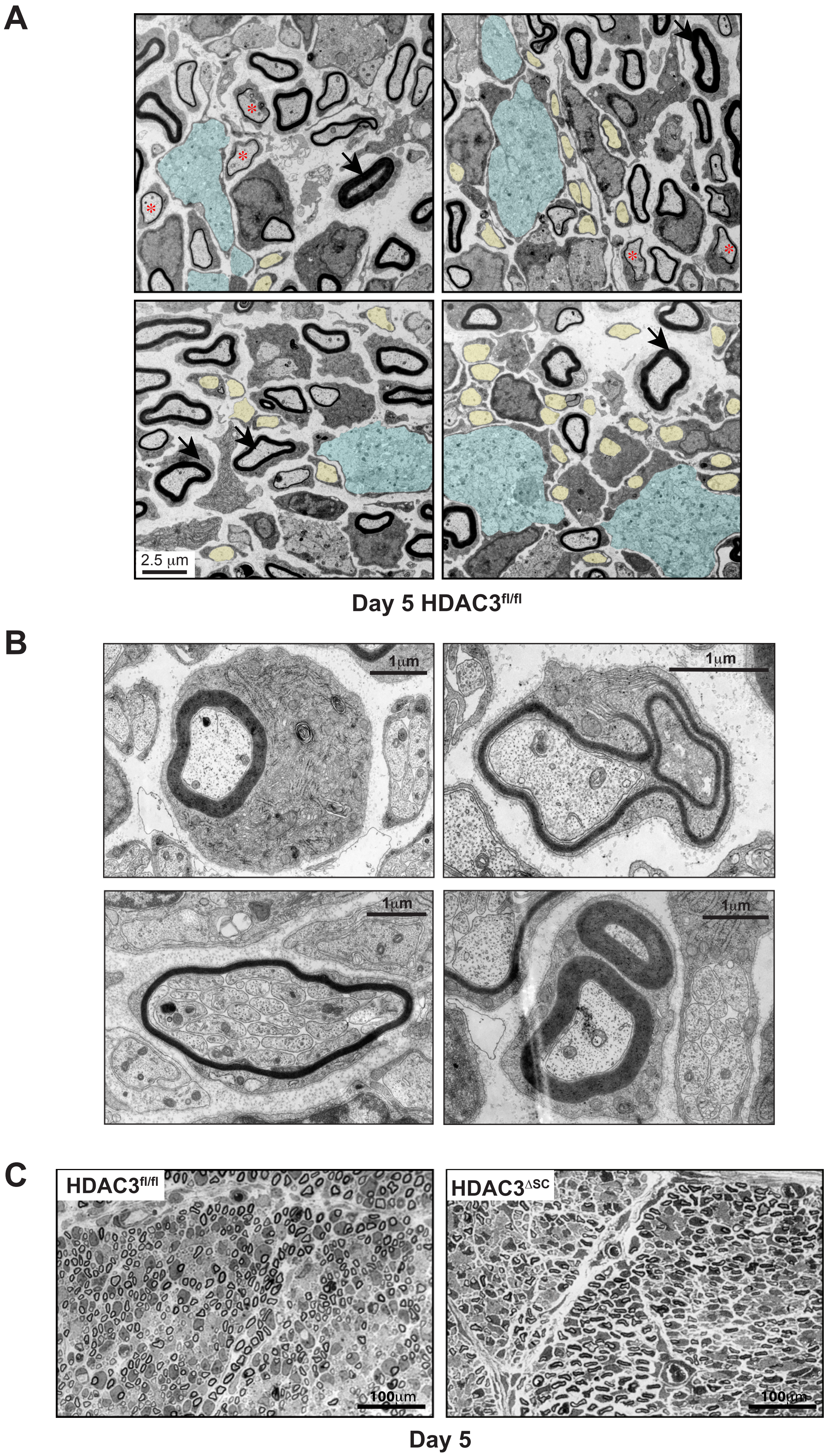
A
2.5 μm
Day 5 HDAC3fl/fl
B
1μm
1μm
1μm
1μm
C
HDAC3fl/fl
HDAC3ΔSC
100μm
100μm
Day 5

Supplementary Figure 4. Myelination initiates normally in Schwann cells lacking HDAC3, related to Figure 4. (A) Representative EM images of transverse ultrathin sections of sciatic nerves from postnatal Day 5 control (HDAC3$^{fl/fl}$) mice. Note that myelination is not a synchronous process: some large and small axons remain unsorted (blue), many Schwann cells have sorted axons and have associated in a 1:1 ratio, but have not yet started the myelination process (yellow), others have just started the myelination process (red *), whereas others have nearly completed myelination (arrows). (B) High magnification EM images of a sciatic nerve from a postnatal Day 5 control HDAC3$^{fl/fl}$ animal showing Schwann cells myelinating normally (top left panel) or abnormally (right top and bottom panels). The bottom left panel shows a Schwann cell myelinating several small diameter axons. C) Representative images of transverse sections of a sciatic nerve from postnatal Day 5 control HDAC3$^{fl/fl}$ and mutant HDAC3$^{\Delta SC}$ animals stained with toluidine blue.

Figure S5

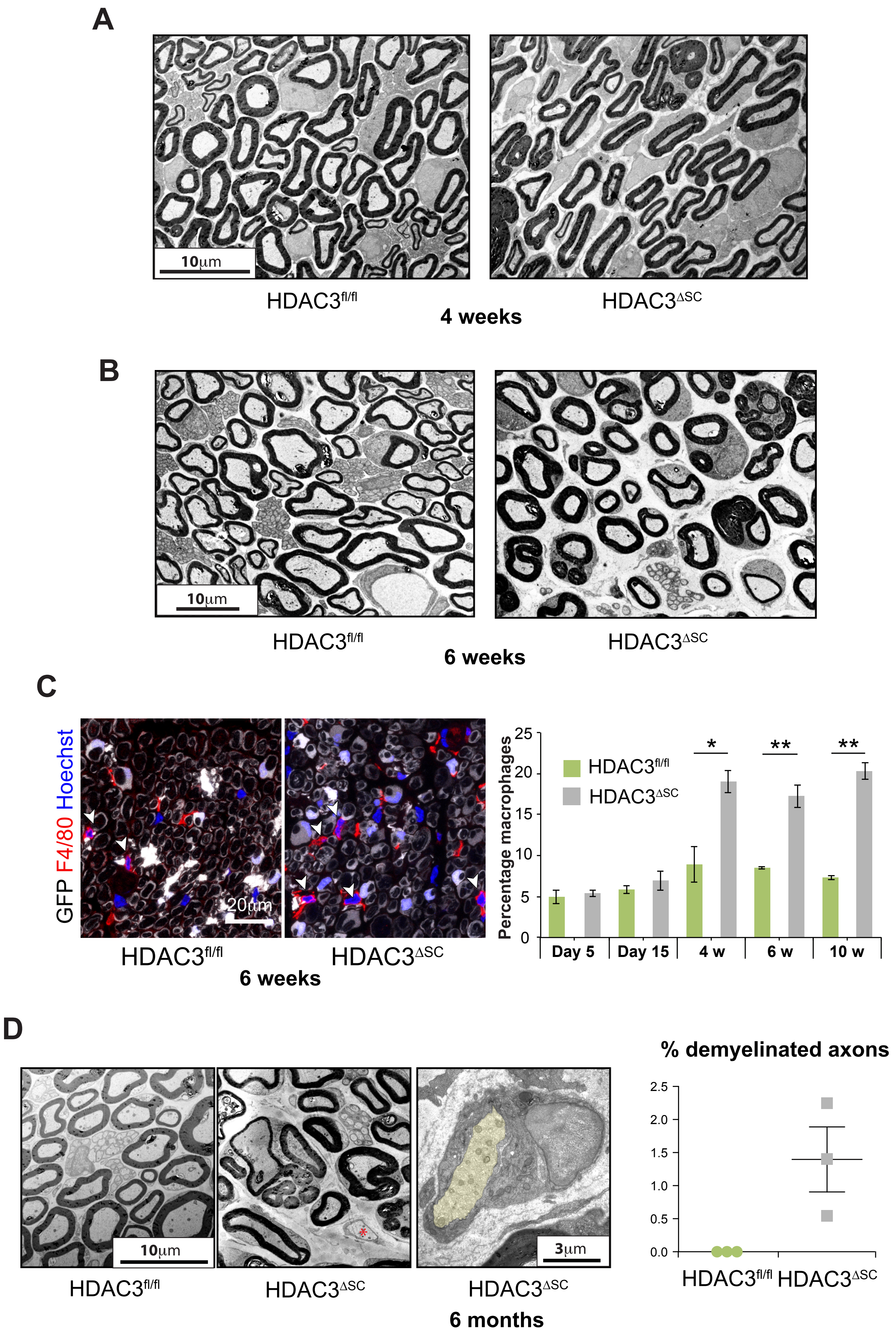

A
10μm
HDAC3fl/fl
HDAC3ΔSC
4 weeks
B
10μm
HDAC3fl/fl
HDAC3ΔSC
6 weeks
C
GFP F4/80 Hoechst
20μm
HDAC3fl/fl
HDAC3ΔSC
6 weeks
Percentage macrophages
25
20
15
10
5
0
HDAC3fl/fl
HDAC3ΔSC
*
**
**
Day 5
Day 15
4 w
6 w
10 w
D
10μm
*
3μm
HDAC3fl/fl
HDAC3ΔSC
HDAC3ΔSC
6 months
% demyelinated axons
2.5
2.0
1.5
1.0
0.5
0.0
HDAC3fl/fl
HDAC3ΔSC

Supplementary Figure 5. Schwann cells lacking HDAC3 fail to enter the homeostatic state, related to Figure 6. (A) and (B) Representative EM images of transverse ultrathin sections of a sciatic nerve from 4 week (A) and 6 week (B) HDAC3$^{fl/fl}$ and mutant HDAC3$^{\Delta SC}$ animals showing worsening of the phenotype overtime. C) Representative immunofluorescent images of transverse sciatic nerve sections labelled to identify macrophages (F4/80, green) and nuclei (Hoescht, blue) in plp-eGFP (SCs GFP+ in white) control (HDAC3$^{fl/fl}$) and mutant mice (HDAC3$^{\Delta SC}$). Graph shows quantification of macrophages at indicated ages in control (HDAC3$^{fl/fl}$) and mutant mice (HDAC3$^{\Delta SC}$) (n=3 or 4 mean±SEM). D) Representative EM images of transverse ultrathin sections of a sciatic nerve from 6 month-old control (HDAC3$^{fl/fl}$) and mutant mice (HDAC3$^{\Delta SC}$) showing a demyelinated axon in mutant mice (HDAC3$^{\Delta SC}$) (red *) (left panel), a high magnification of a demyelinated axon (yellow) found in a mutant mouse (HDAC3$^{\Delta SC}$) (middle panel) and the quantification of demyelinated axons at that age (n=3 mean±SEM). Note that we did not find any demyelinated axons in the control animals. *$p<0.05$, **$p<0.01$.

Movie S2, related to Figure 5. mSCs lacking HDAC3 develop normally. Movie shows serial EM images of entire mSCs in postnatal Day 15 control (HDAC3$^{fl/fl}$) and mutant (HDAC3$^{\Delta SC}$) mice. Serial 70nm ultrathin longitudinal sections of sciatic nerves were imaged and then aligned. Black arrowheads indicate the mSCs that were used to generate the 3D-reconstructions shown in Figure 5E.

Figure S6

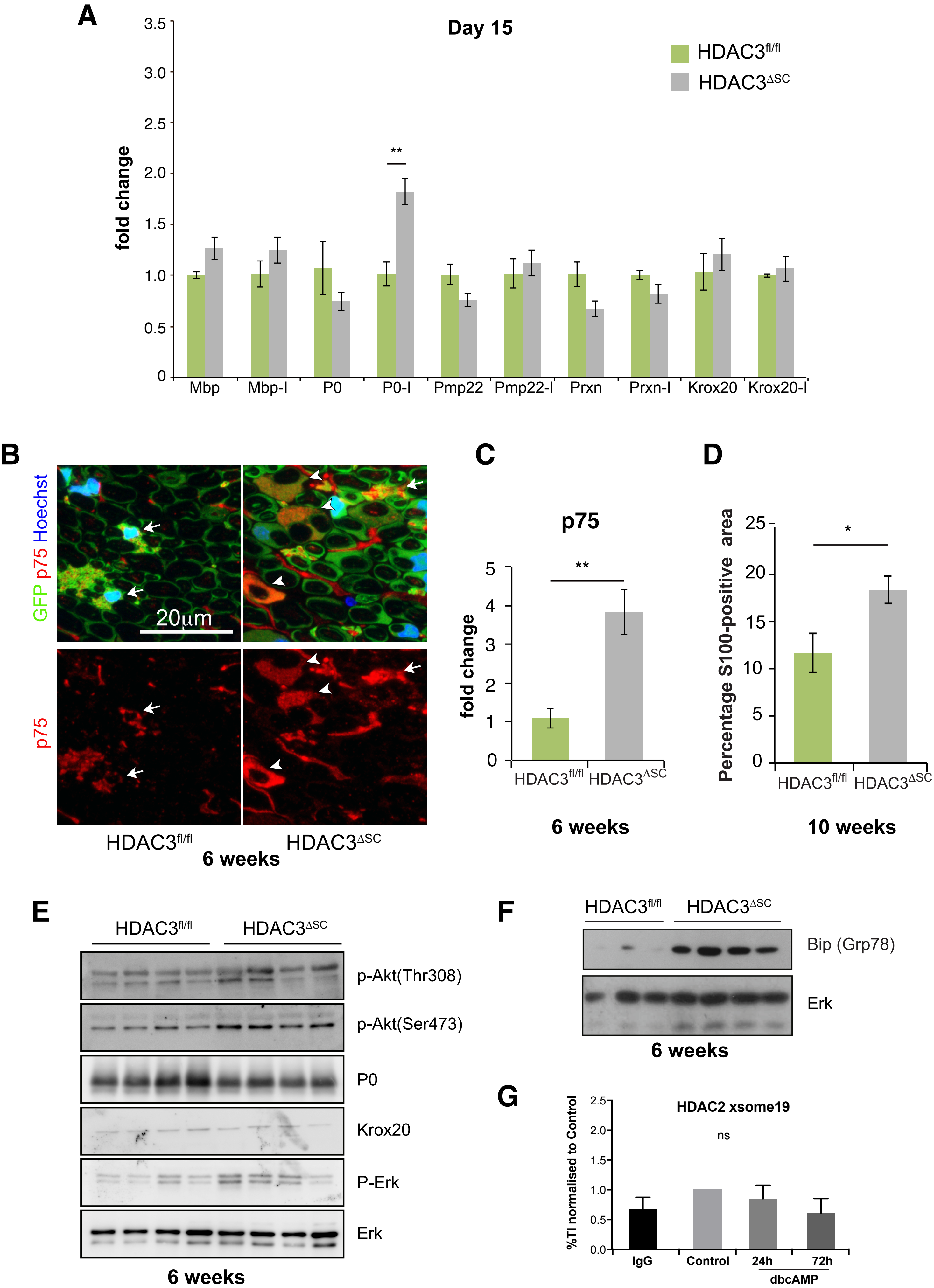
A
Day 15
HDAC3fl/fl
HDAC3ΔSC
fold change
3.5
3.0
2.5
2.0
1.5
1.0
0.5
0
**
Mbp
Mbp-I
P0
P0-I
Pmp22
Pmp22-I
Prxn
Prxn-I
Krox20
Krox20-I
B
GFP p75 Hoechst
p75
20μm
HDAC3fl/fl
HDAC3ΔSC
6 weeks
C
p75
fold change
**
HDAC3fl/fl
HDAC3ΔSC
6 weeks
D
Percentage S100-positive area
*
HDAC3fl/fl
HDAC3ΔSC
10 weeks
E
HDAC3fl/fl
HDAC3ΔSC
p-Akt(Thr308)
p-Akt(Ser473)
P0
Krox20
P-Erk
Erk
6 weeks
F
HDAC3fl/fl
HDAC3ΔSC
Bip (Grp78)
Erk
6 weeks
G
HDAC2 xsome19
ns
%TI normalised to Control
IgG
Control
24h
72h
dbcAMP

Supplementary Figure 6. Myelinating Schwann cells lacking HDAC3 remain in the biogenic state, related to Figure 7. A) RT-qPCR analysis of key myelin genes and the Krox-20 transcription factor in postnatal Day 15 HDAC3$^{fl/fl}$ and mutant HDAC3$^{\Delta SC}$ mice. Graph shows the mRNA or nascent pre-mRNA (-I) levels of mutant sciatic nerves relative to the controls (n = 3 for HDAC3$^{fl/fl}$, n=4 for HDAC3$^{\Delta SC}$, 3 animals were pooled for each sample, mean ± SEM). B). Representative immunofluorescent images of transverse sciatic nerve sections labelled with p75 (red) to identify non-myelinating Schwann cells (arrows) and mSCs that have dedifferentiated (arrowheads) in 6 weeks old plp-eGFP (GFP+ SCs in green) control (HDAC3$^{fl/fl}$) and mutant mice (HDAC3$^{\Delta SC}$). Nuclei were counterstained with Hoechst (blue). C) Graph shows p75 mRNA levels of mutant sciatic nerves relative to the controls at 6 weeks (n = 4 for HDAC3$^{fl/fl}$, n=4 for HDAC3$^{\Delta SC}$, 2 animals were pooled for each sample, mean±SEM) D) Quantification of Figure 7B showing the proportion of S100-positive area in 10 week-old HDAC3$^{fl/fl}$ and HDAC3$^{\Delta SC}$ animals. Note that the cytoplasm of Schwann cells is significantly enlarged in mSCs lacking HDAC3 (n=3 mean±SEM). E) Western blot analysis of total protein lysates from 4 week-old HDAC3$^{fl/fl}$ and mutant HDAC3$^{\Delta SC}$ mice showing no changes between the two genotypes in signalling through the ERK or PI-3kinase pathways. F) Western blot analysis of total protein lysates from 6 week-old HDAC3$^{fl/fl}$ and HDAC3$^{\Delta SC}$ mice showing an upregulation of the molecular chaperone protein BiP/Grp78 in mutant HDAC3$^{\Delta SC}$ mice. G) ChIP analysis of HDAC2 on the negative control region (gene desert on Chromosome 19), in NSΔRafER cells cultured with ± dbcAMP for the indicated times (n = 4, mean±SEM). Note that the % total input of each sample was lower than 0.1% indicating there was no binding on this DNA region. *p<0.05, **p<0.01.

Table S1 Primer Sequences for qPCR, related to STAR methods

	Forward 5'-3'	Reverse 5'-3'
Mouse myelin binding protein (mbp) mRNA	gaagctcgtcggactctgag	ggcggtgacagactccaag
Mouse myelin protein zero (P0) mRNA	cggacagggaaatctatggtgc	tggtagcgccaggtaaaagag
Mouse peripheral myelin protein 22 (pmp22) mRNA	catcgcggtgctagtgttg	aaggcggatgtggtacagttc
Mouse periaxin (prxn) mRNA	tcagcggcttcaacgtagc	tagctgccggtgagtcctc
Mouse early growth response protein 2 (Krox20) mRNA	gccaaggccgtagacaaaatc	ccactccgttcatctggtca
Mouse myelin binding protein (mbp) pre-mRNA	caaagcgcaaaagttcccga	cgtgggatacacgagacctg
Mouse myelin protein zero (P0) pre-mRNA	gagattcgggacaatgagggg	aggaggaggcaaccaaacaa
Mouse peripheral myelin protein 22 (pmp22) pre-mRNA	tgttgctgcacttctttgcc	ccttgctcactgtctacccc
Mouse early growth response protein 2 (Krox20) pre-mRNA	atcgccccaaagtgaacagg	cctaggcattctccttgccg
Mouse p75 neurotrophine receptor (NTR) mRNA	acattccgaccgctgatgtt	gacacagaggccctacacag
Mouse Histone deacetylase 3 (hdac3) mRNA	aatgtgcccttacgagatgg	gtagccaccacctcccagta
Mouse beta-2 microglobulin (b2m) mRNA	ttctggtgcttgtctcactga	cagtatgttcggcttcccattc
Rat myelin protein zero (P0) mRNA	ctggtccagtgaatgggtct	catgtgaaagtgccgttgtc
Rat myelin protein zero (P0) pre-mRNA	gaccatatttggcaaggggc	gggtgtcgtctcacgtagc
Rat beta-2 microglobulin (b2m) mRNA	tgaccgtgatctttctggtg	atttgaggtgggtggaactg

Table S2 Primer Sequences for ChIP, related to STAR methods

	Forward 5'-3'	Reverse 5'-3'
P0 enhancer	gtagttatgagcccccagca	tcaccctctccttggtcatc
P0 promoter	tttctgtccctctgcctcac	gtcctgagccagtgaccaa
Xsome19 gene desert	agcctactttcttggcttgc	atgaagctgggctagcagat
