# Supplementary material for: HDAC3 Regulates the Transition to the Homeostatic Myelinating Schwann Cell State
Source: Cell Rep. 2018 Dec 4;25(10):2755–2765.e5. doi: 10.1016/j.celrep.2018.11.045 (PMC6293966; doi:10.1016/j.celrep.2018.11.045)
Supplement: Document S2. Article plus Supplemental Information [file mmc4.pdf]

Cell Reports

Article

HDAC3 Regulates the Transition to the Homeostatic Myelinating Schwann Cell State

Graphical Abstract

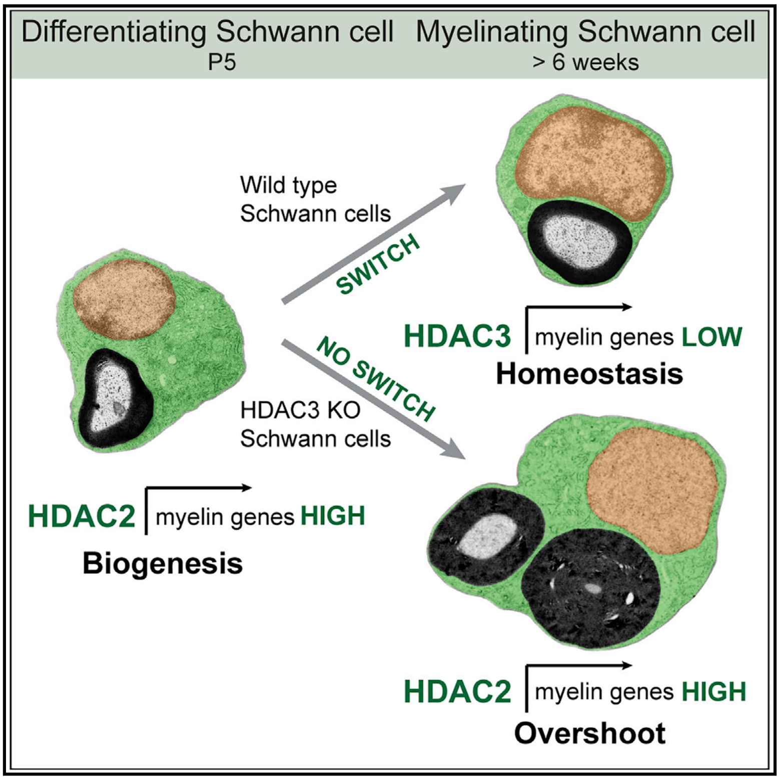

Highlights

- HDAC3 is a positive regulator of myelin gene expression
- Loss of HDAC3 expression in Schwann cells results in severe neuropathies
- Myelination proceeds normally without HDAC3 but "overshoots" in adult mice
- HDAC3 is required for myelinating Schwann cells to enter the homeostatic state

Authors

Laura H. Rosenberg, Anne-Laure Cattin, Xavier Fontana, ..., Robin Ketteler, Antonella Riccio, Alison C. Lloyd

Correspondence

alison.lloyd@ucl.ac.uk

In Brief

The entry of differentiating cells into a homeostatic state is poorly understood. Here, Rosenberg et al. show that a switch between HDAC1/2 and HDAC3 is responsible for the entry of myelinating Schwann cells into homeostasis with HDAC3$^{-/-}$ Schwann cells myelinating normally but "overshooting," resulting in severe neuropathies in adult mice.

Rosenberg et al., 2018, Cell Reports *25*, 2755–2765
December 4, 2018 © 2018 The Author(s).
https://doi.org/10.1016/j.celrep.2018.11.045

CellPress

OPEN ACCESS
CellPress

HDAC3 Regulates the Transition to the Homeostatic Myelinating Schwann Cell State

Laura H. Rosenberg,[1,3,6] Anne-Laure Cattin,[1,6] Xavier Fontana,[1] Elizabeth Harford-Wright,[1] Jemima J. Burden,[1] Ian J. White,[1] Jacob G. Smith,[1] Ilaria Napoli,[1] Victor Quereda,[1,5] Cristina Policarpi,[1] Jamie Freeman,[1,4] Robin Ketteler,[1] Antonella Riccio,[1] and Alison C. Lloyd[1,2,7,*]
[1]MRC Laboratory for Molecular Cell Biology, University College London, Gower Street, London WC1E 6BT, UK
[2]UCL Cancer Institute, University College London, Gower Street, London WC1E 6BT, UK
[3]CRUK Therapeutic Discovery Laboratories, Babraham Research Campus, Cambridge CB22 3AT, UK
[4]Horizon Discovery, 8100 Cambridge Research Park, Cambridge CB25 9TL, UK
[5]The Scripps Research Institute, 130 Scripps Way, Jupiter, FL 33458, USA
[6]These authors contributed equally
[7]Lead Contact
*Correspondence: alison.lloyd@ucl.ac.uk
https://doi.org/10.1016/j.celrep.2018.11.045

SUMMARY

The formation of myelinating Schwann cells (mSCs) involves the remarkable biogenic process, which rapidly generates the myelin sheath. Once formed, the mSC transitions to a stable homeostatic state, with loss of this stability associated with neuropathies. The histone deacetylases histone deacetylase 1 (HDAC1) and HDAC2 are required for the myelination transcriptional program. Here, we show a distinct role for HDAC3, in that, while dispensable for the formation of mSCs, it is essential for the stability of the myelin sheath once formed—with loss resulting in progressive severe neuropathy in adulthood. This is associated with the prior failure to downregulate the biogenic program upon entering the homeostatic state leading to hypertrophy and hypermyelination of the mSCs, progressing to the development of severe myelination defects. Our results highlight distinct roles of HDAC1/2 and HDAC3 in controlling the differentiation and homeostatic states of a cell with broad implications for the understanding of this important cell-state transition.

INTRODUCTION

Myelinating Schwann cells (mSCs) are critical for the function of the peripheral nervous system (PNS) providing both a nurturing function to axons and the periodic insulation essential for efficient saltatory conduction (Salzer, 2015). The mSC is first specified before birth, during the axonal organization process known as radial sorting, in which progenitor Schwann cells identify axons larger than 1 μm in diameter associate in a 1:1 ratio and in response to axonal signals exit the cell cycle and start to express transcription factors specific to the myelinating cell type (Jessen and Mirsky, 2005; Monk et al., 2015). Myelination itself is initiated in the early post-natal period and is an extraordinary biogenic process involving a several thousand-fold expansion in the specialized membrane that forms the myelin sheath (Garbay et al., 2000). Once this process is complete, the mSC transitions to the homeostatic state that can be maintained for the lifespan of the animal. This requires the continued expression of myelin genes but at the lower levels necessary for the maintenance of the sheath (Decker et al., 2006; Salzer, 2015; Toyama et al., 2013). The switch from a construction "biogenic" state to a maintenance/homeostatic state, and the nature of the stability of the homeostatic state, is likely to be important for many non-dividing, long-lived cells in the body, but how these processes are controlled remains poorly understood (Lloyd, 2013; Roberts and Lloyd, 2012).

What is clear is that exquisite control of the transcriptional regulation of the myelinating state is critical for the function of the mSC (Nave et al., 2007; Pereira et al., 2012). In mice, the myelination transcriptional program is initiated during development in response to axonal signals and involves a transcriptional feedforward network that ultimately leads to the expression of the master transcriptional regulator of myelination, Krox-20, and the onset of myelination in the post-natal period (Pereira et al., 2012; Stolt and Wegner, 2016; Topilko et al., 1994). The Krox-20-dependent production of the myelin sheath requires the rapid, extensive production of lipids and myelin proteins yet the stability of this process is extremely sensitive to the stoichiometry of its protein components (Camargo et al., 2009; D'Antonio et al., 2013; Nave and Werner, 2014; Topilko et al., 1994). This has been most clearly demonstrated by genetic neuropathies in which an additional copy of a myelin gene is sufficient to induce a severe neuropathy (Nave et al., 2007). Once the mSC is formed, the level of myelin gene transcription drops to lower levels, but active myelin gene transcription is still required in the adult, as shown by studies showing that Krox-20 deletion in the adult resulted in demyelination (Decker et al., 2006). These studies demonstrate that Krox-20-dependent transcription is required for both the differentiation and maintenance of the myelinated state but implies that additional regulatory processes must control the levels of transcription.

This is an open access article under the CC BY license (http://creativecommons.org/licenses/by/4.0/).

OPEN ACCESS
CellPress

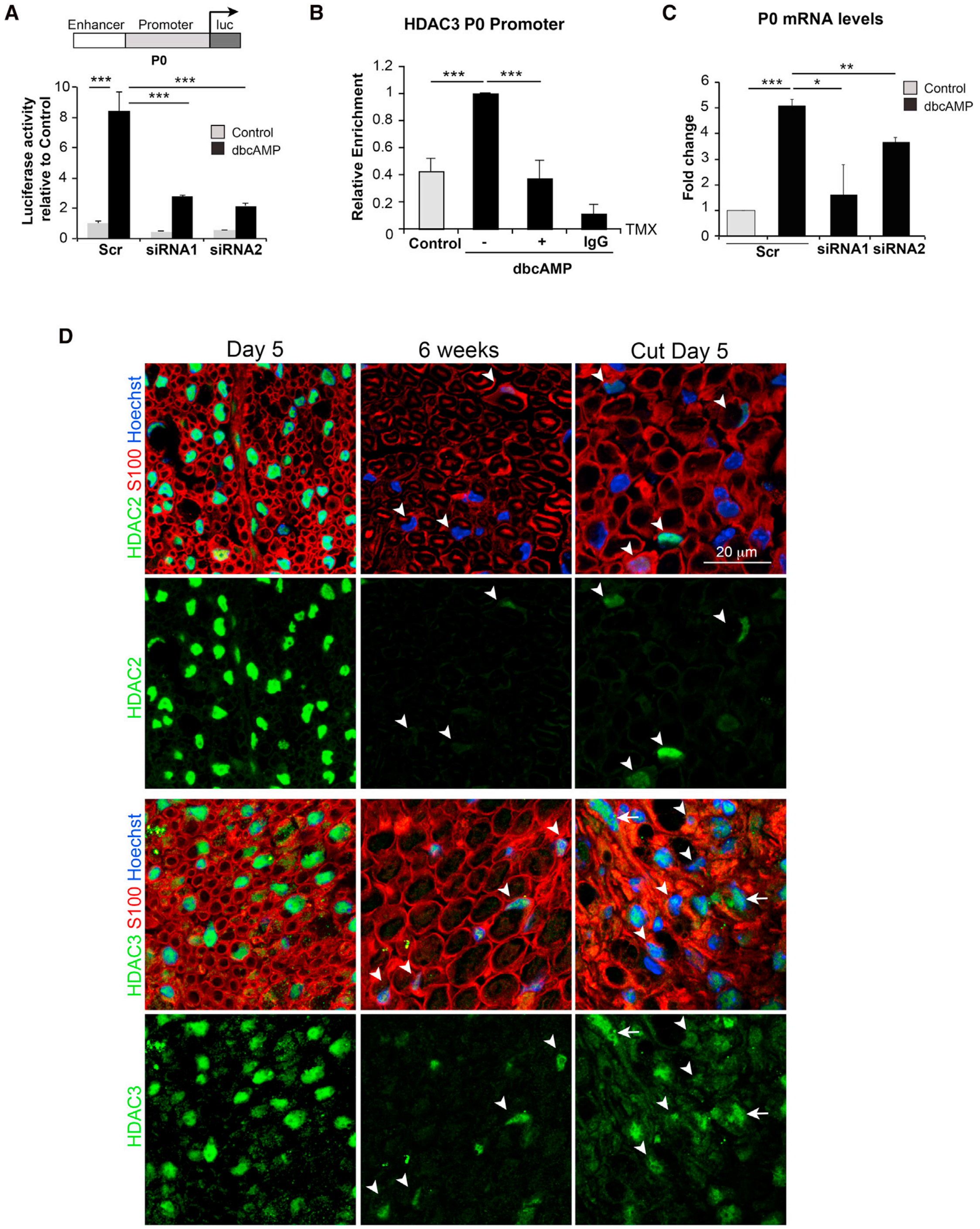

Figure 1. HDAC3 Regulates Myelin Gene Transcription and Is Expressed in Adult Myelinating Schwann Cells

(A) Relative luciferase activity of the regulatory elements of the P0 gene (promoter plus enhancer; see STAR Methods for further details) in the absence (control) or presence (dbcAMP) of dbcAMP for 24 hr following the transfection of scrambled (Scr) or two independent siRNAs (siRNA1 and siRNA2) (n = 3, mean ± SEM).

(B) ChIP analysis to detect HDAC3 binding to the P0 promoter. SCs expressing a tamoxifen (TMX)-inducible Raf kinase construct (NSΔRafER cells) were cultured in the absence of presence of dbcAMP for 72 hr and then for a further 24 hr in the absence or presence (−/+) of TMX to induce the dedifferentiation of the cells (n = 3, mean ± SEM).

(C) Relative endogeneous P0 mRNA levels following transfection of scrambled (Scr) or two independent siRNAs (siRNA1 and siRNA2) in the absence (control) or presence (dbcAMP) of dbcAMP (n = 3, mean ± SEM).

(legend continued on next page)

Histone deacetylases (HDACs) are a large family of proteins that function as transcriptional regulators and control gene expression mainly by modulating the acetylation levels of histones with resulting effects on chromatin compaction (Haberland et al., 2009). HDACs were initially thought to act mainly as transcriptional repressors and were found in large multiprotein complexes with transcriptional co-repressors. However, numerous more recent studies have shown that subsets of genes require HDAC activity for their expression (Nott et al., 2016; Wang et al., 2009; Zupkovitz et al., 2006). Furthermore, HDACs have non-histone transcriptional targets and can exert some of their functions independently of their enzymatic activity, suggestive of more complex roles in regulating the multiprotein complexes controlling transcription (Seto and Yoshida, 2014).

The class 1 HDACs, HDAC1, and HDAC2 and the associated members of the NuRD complexes have been shown to play an important role in regulating Schwann cell (SC) myelination (Brügger et al., 2017; Hung et al., 2012; Jacob, 2017; Jacob et al., 2011; Quintes et al., 2016; Wu et al., 2016). SC-specific double knockout of HDAC1 and HDAC2 or knockout of NuRD components leads to defects in SC myelination with the complexes required both for the repression of progenitor genes and the expression of the myelin gene program. Here, in a non-biased screen, we identified HDAC3 as a regulator of myelin gene expression. In contrast to HDAC1/2, we found that HDAC3 was not required for the development of the myelinating cell but was instead critical for the entry into and the maintenance of the homeostatic state. These findings provide insight into the mechanisms that can govern the transition into the homeostatic state and have implications for the understanding of disorders such as neuropathies and aging.

RESULTS

A Non-biased siRNA Screen Identifies HDAC3 as a Regulator of Myelination

In order to identify transcriptional regulators of myelination, we conducted a non-biased small interfering RNA (siRNA) screen of chromatin regulators in primary SCs expressing a luciferase reporter under the control of a well-characterized promoter-enhancer region of the myelin protein zero (mpz) gene (P0) (LeBlanc et al., 2006). We identified HDAC3 as a potential regulator of myelination and validation of the screen confirmed that HDAC3 was a positive regulator of the P0 transcriptional regulatory elements (Figures 1A and S1A). Chromatin immunoprecipitation (ChIP) analysis confirmed that HDAC3 was found at the P0 promoter in differentiated mSCs (Figure 1B). Moreover, knockdown of HDAC3 in an *in vitro* differentiation assay confirmed that HDAC3 is a positive regulator of myelin gene expression (Figure 1C).

HDAC1 and HDAC2 have been shown to be expressed in SCs during development and to be essential for SC myelination that takes place in the early post-natal period (Jacob, 2017; Jacob et al., 2011). In adulthood, HDAC1/2 expression levels decrease dramatically and the lower levels of HDAC2 appear to have a distinct role in the adult in controlling paranodal and nodal stability (Brügger et al., 2015). However, HDAC1/2 levels increase following injury as SCs return to a progenitor-like state consistent with a role in the control of progenitor SC function (Jacob et al., 2011). Notably, we found that HDAC3 had a very distinct pattern of expression. Similarly to HDAC2, HDAC3 expression was readily observed in the nuclei of mSCs at postnatal day 5; however, in contrast to HDAC2, HDAC3 expression was maintained in the adult in both mice and rats (Figures 1D and S1B). Moreover, HDAC3 levels decreased following injury suggesting distinct roles for HDAC1/2 and HDAC3 in regulating SC behavior.

Loss of HDAC3 in Schwann Cells Results in a Progressive Adult Neuropathy

In order to investigate the function of HDAC3 in SCs, we knocked out HDAC3 specifically in SCs by crossing mice carrying a floxed allele of HDAC3 (Montgomery et al., 2008) to mice expressing Cre recombinase under the control of the P0 promoter (P0:HDAC3$^{fl/fl}$) (Feltri et al., 2002). This well-characterized promoter drives the expression of Cre in SCs from around embryonic day 13.5, which is prior to SC driven axonal sorting or the differentiation of SCs into either myelinating or non-myelinating (Parrinello and Lloyd, 2009). Consistent with this, we found that HDAC3 was efficiently (87.4% ± 4.6%) deleted from SCs during development (Figures 2A and S2A–S2C) as determined by immunostaining, whereas HDAC3 levels remained unchanged in other HDAC3-expressing cells, such as endothelial cells and macrophages, within the nerve (Figure S2D).

Mutant mice developed normally and initially showed no apparent abnormalities. However, upon reaching 2 months of age, the mice began to develop motor deficits and with age these deficiencies worsened to include severe weakness, limb clasping, claw toe, and muscle wastage, especially in their rear quarters (Figures 2B, 2C, S2E, and S2F; Video S1). These are features that resemble Charcot-Marie-Tooth (CMT) syndromes in humans (Vallat et al., 2013) and indicate that HDAC3 expression in SCs is required for proper nerve function.

In order to investigate the pathology of the sciatic nerves, the nerves were analyzed at 36 weeks, when the phenotype was severe. Toluidine-stained semi-thin sections showed gross abnormalities of the nerves consistent with the behavioral deficits. The defects varied from severe myelination abnormalities to regions in the distal portion of some nerves in which nearly all axons appeared lost and the tissue appeared fibrotic with abundant extracellular matrix (Figure S3A). Ultrathin electron microscopy (EM) images from the same mice showed that loss of HDAC3 caused a variety of gross abnormalities in nearly all of the SC-axonal units (Figures 3A and

(D) Representative confocal images of mouse sciatic nerve of postnatal P5, 6-week-old animals, and 6-week-old animals, 5 days following transection stained for HDAC3 or HDAC2 (green) as indicated with SCs labeled for S100 (red). Note that whereas HDAC2 expression in adulthood is at low levels in myelinating Schwann cells (mSCs) (arrowheads), it is re-induced upon injury (arrowheads). Conversely, nuclear HDAC3 expression is maintained in adult mSCs (arrowheads), whereas it decreases upon injury in myelinating-derived SCs (arrowheads). Other cell types express high levels of HDAC3 after injury (arrows).

*p < 0.05, **p < 0.01, ***p < 0.001. See also Figure S1.

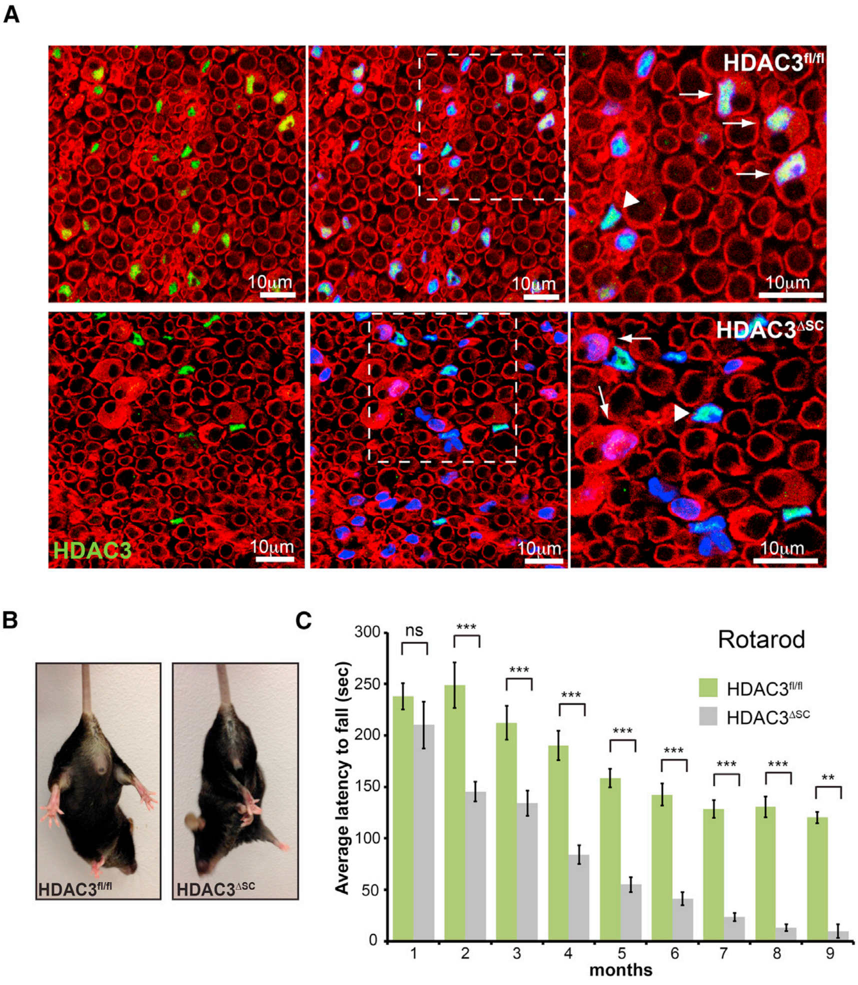

Figure 2. Loss of HDAC3 in Schwann Cells Results in Progressive Adult Neuropathy

(A) Immunofluorescence of representative transverse sciatic nerve sections from control (HDAC3$^{fl/fl}$) mice at postnatal day 15 or mutant mice (HDAC3$^{\Delta SC}$) showing efficient loss of nuclear HDAC3 staining (green) in S100-labeled SCs (red). Nuclei were counterstained with Hoechst (blue). Arrows point to mSCs and arrowheads to other cell types that also express HDAC3.

(B) Images of 6-month-old HDAC3$^{fl/fl}$ and HDAC3$^{\Delta SC}$ animals showing hind limb clasping and muscle wastage.

(C) Rotarod behavioral tests showing average latency to fall of control HDAC3$^{fl/fl}$ and mutant HDAC3$^{\Delta SC}$ animals from 1 to 9 months after birth (n = 4–19 mean ± SEM).

**$p < 0.01$, ***$p < 0.001$. See also Figure S2 and Video S1.

S3B and quantified in Figure 3B). Notably, myelin sheaths were grossly affected and displayed dramatic myelin outfoldings that have been associated with myelin dysregulation, usually overproduction (Adlkofer et al., 1997; Bolis et al., 2005; Tersar et al., 2007). A wide variety of defects were observed including gross hypermyelination of individual axons, myelination of more than one axon by an individual SC, and myelin outfoldings into the axon (Figures 3C and S3C). Interestingly, despite these severe myelin abnormalities, the compaction and structure of the myelin appeared normal within the outfoldings (Figure S3B). Myelin degeneration was common and many axons had lost their myelin sheath. This was accompanied by loss of axons, suggesting that secondary to the SC myelination defects there was also loss of neuronal fibers (Figures 3A, 3B, and S3B).

mSCs Lacking HDAC3 Fail to Enter into the Adult Homeostatic State

To determine the first manifestations of the phenotype, we examined nerves from mice at earlier ages. At postnatal day 5, myelination is underway; however, it does not proceed synchronously in that, at this time point, there is a mixture of axons that are myelinating, others that remain unsorted within axonal bundles and those that have been sorted into a 1:1 ratio with a SC but myelination has not yet initiated (Figures 4A and S4A). Consistent with the highly biogenic state of the mSCs at this age, they resemble "factories" with a large cytoplasm full of ER, Golgi, and mitochondria (Figure S4B).

Interestingly, we found that, in contrast to HDAC1/2 mutant mice, nerves apparently developed normally in HDAC3 mutant mice with nerves from control and mutant mice visibly indistinguishable from each other (Figure 4A) with normal g-ratios indicating the mutant nerves develop normally with SCs lacking HDAC3 (Figures 4A, 4B, and S4C). However, quantification revealed a small decrease in the number of myelinated axons per field and slightly larger numbers of unsorted axons consistent with a minor delay in the myelination process; moreover, a slight increase in axonal diameter was also observed (Figures 4C–4E). This suggests that the mutant mice may have a minor sorting defect that could affect the growth of the axons, but the mildness of the phenotype makes this difficult to interpret. Interestingly at this stage, we observed a number of defects in the control animals with bundles of small axons being myelinated by a single SC and abnormal outfoldings of myelin (Figures S4B) indicating that myelination is not a perfect process during development. Overall, these results indicate that while HDAC1/2 are required for myelination, HDAC3 ablation in SCs has a minimal effect on these processes, showing that HDAC3 is not required for SC myelination or for the correct formation of Remak bundles.

By P15, myelination is more complete with some mSCs appearing to have finished the process. However, even at this age, myelination has not been initiated in some mSCs, whereas, in many others, the myelination process is still in progress

OPEN
ACCESS
CellPress

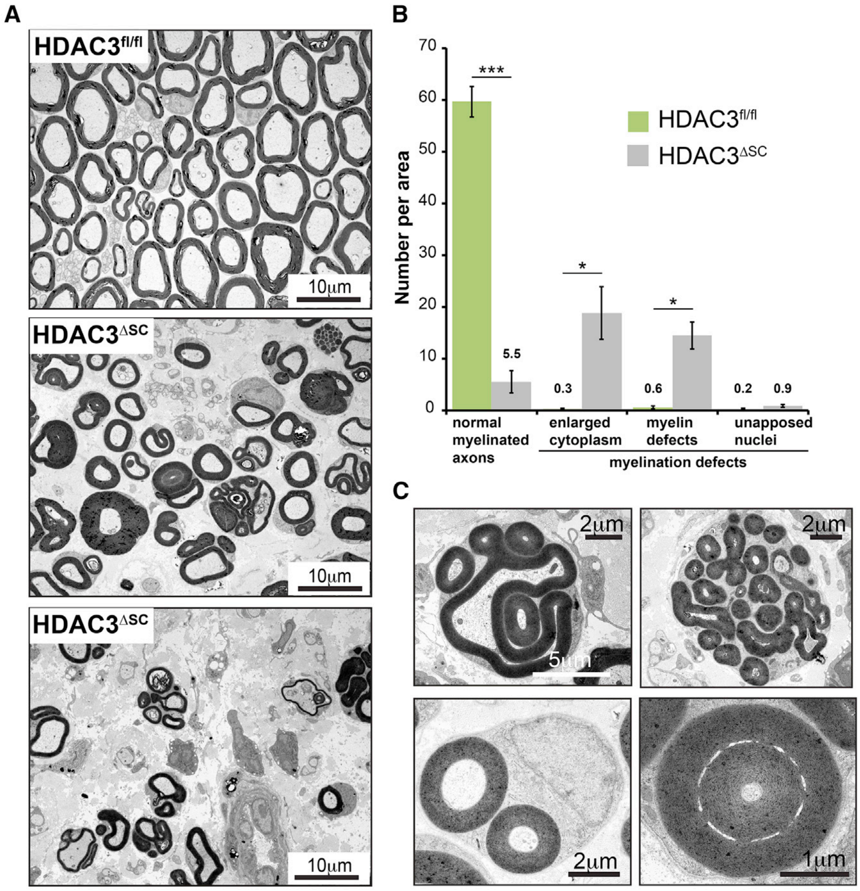

9 months

Figure 3. HDAC3 Loss in Schwann Cells Results in Gross Myelinating Abnormalities

(A) Representative EM images of transverse ultrathin sections of sciatic nerves from 9-month-old HDAC3$^{fl/fl}$ and HDAC3$^{\Delta SC}$ animals, when the mutant animals exhibit profound neuropathies. The two images from the mutant animals represent areas of less severe and severe myelination defects.

(B) Quantification of myelination defects in 9-month-old animals (n = 3 mean ± SEM).

(C) Selected images of myelination abnormalities including myelin outfoldings (top left), focal hypermyelination (top right), the myelination of two axons by a single SC (bottom left), and redundant loop formation (bottom right).

*$p < 0.05$, ***$p < 0.001$. See also Figure S3.

(Figures 5A and 5B). By this age, however, we were able to clearly distinguish mutant from littermate control nerves in that hypermyelination was observable in a small minority of SC:axonal units (Figures 5A and 5B and quantified in Figure 5C). The mildness of the phenotype was reflected by g-ratio analysis, which showed that whereas the g-ratios were similar for the majority of axons in control and mutant animals, a few axons in the mutant animals showed lower g-ratios consistent with hypermyelination (Figure 5D). Moreover, we performed 3D reconstructions of longitudinal EM sections of sciatic nerves to visualize defects throughout the cells. This analysis confirmed the mildness of the phenotype in that of five randomly selected mSCs from control and mutant mice only one of each genotype showed abnormalities in the myelin sheath (Figure 5E; Video S2; data not shown).

As the animals aged, we observed that the number of abnormalities progressively increased (Figures 6A–6C, S5A, and S5B) with altered g-ratios observable by 4 weeks (Figure 6C). By 10 weeks, the vast majority of mSCs (>80%) showed gross abnormalities consistent with the age at which the first motor abnormalities were observed (Figures 2B, 6D, and 6E). A notable abnormality was that many of the mSCs displayed a massively enlarged cytoplasm and the nucleus failed to appose to the myelin sheath in the majority of the cells (62.87% ± 9.97% SEM in mutant mice at 6 weeks versus 0% in controls), a characteristic position in normal mature mSCs, which are highly polarized (Figures 6D–6F). In addition, many of these enlarged cells became filled with comma-shaped myelin sheath outfoldings and showed gross myelin over-production. Consistent with this abnormal nerve environment, an inflammatory response starting at 4 weeks was observed with an increased number of macrophages found within mutant nerves (Figure S5C). By 6 months, when the behavioral phenotype starts to become more severe, a few axons showed complete demyelination (Figure S5D), which progressed to the severe axonal loss seen at 9 months (Figures 3A–3C and S3A–S3C).

Schwann Cells Lacking HDAC3 Remain in the Biogenic Phase of Myelination

Between 2 and 4 weeks, as the myelination process passes its peak, the high levels of myelin production required for the formation of the myelin sheath drop to the levels needed to sustain the myelin sheath during adulthood (Garbay et al., 2000). This switch from the differentiation to the adult homeostatic state is also associated with a characteristic morphological change as the nucleus tightly apposes to the sheath and the cytoplasm is restricted to a thin ribbon surrounding the sheath (Figure 6D; Garbay et al., 2000; Nave and Werner, 2014). This highly polarized structure is thought to be important for the function of the mSC both to nurture the axon and to provide the stable insulation required for efficient saltatory conduction (Nave, 2010; Nave and Werner, 2014). This led us to hypothesize that, in HDAC3 mutant mice, this switch to the homeostatic state was failing to take place and that the mSCs were continuing to produce myelin at rates associated with the differentiation stage. Consistent with this, we found that HDAC3 mutant mice nerves at 6 weeks were transcribing

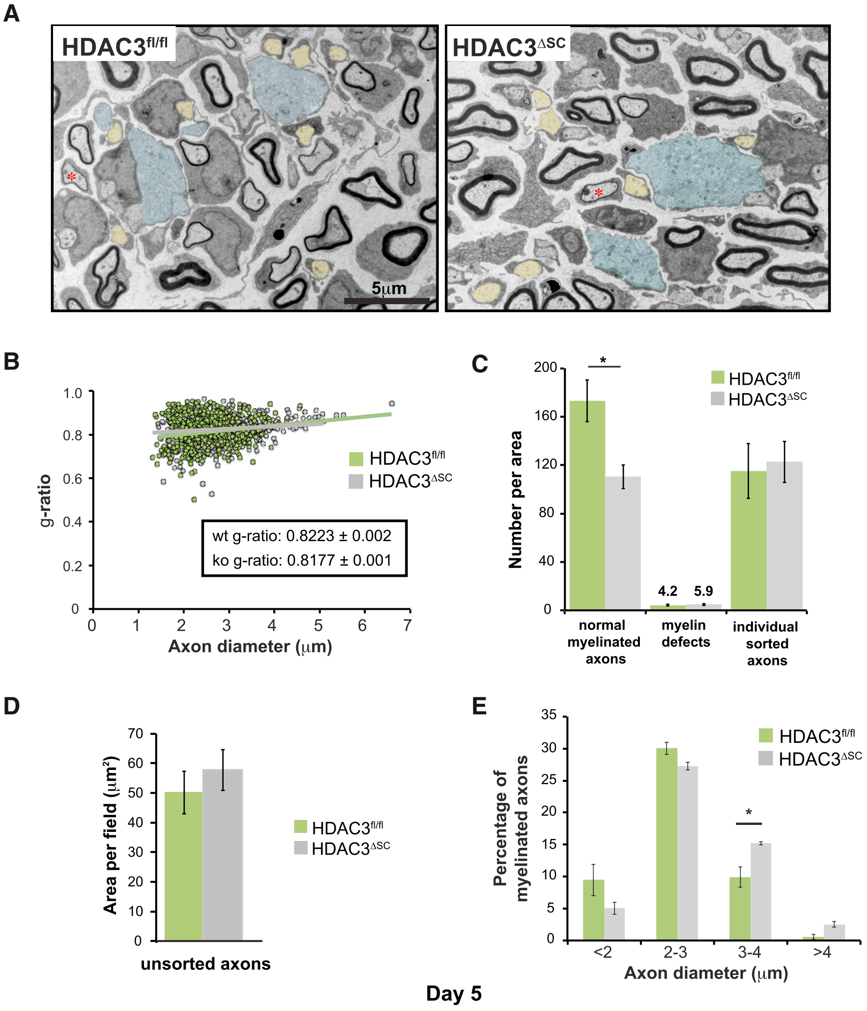

Figure 4. Myelination Initiates Normally in Schwann Cells Lacking HDAC3

(A) Representative colored EM images of sciatic nerve sections from postnatal day 5 animals. Unsorted axons are shown in blue, single sorted axons prior to myelination are shown in yellow, and sorted axons that have just initiated myelination are indicated with a red asterisk.

(B) Graph shows the g-ratio as a function of axon diameter of sciatic nerves from HDAC3$^{fl/fl}$ and HDAC3$^{\Delta SC}$ postnatal day 5 mice (n = 3 > 600 axons/genotype).

(C–E) Graphs show (C) quantification of the myelination process as indicated, (D) the overall area of unsorted axons, and (E) the axon diameters of mSCs in the sciatic nerves of postnatal day 5 HDAC3$^{fl/fl}$ and HDAC3$^{\Delta SC}$ mice (n = 3 mean ± SEM).

*$p < 0.05$. See also Figure S4.

much higher levels of myelin genes than control mice, whereas, at early times during the peak of myelin production, the levels were similar in the HDAC3 mutant and control mice (Figures 7A and S6A). We did not observe increased transcription of all of the myelin genes but think this is likely due to the lack of synchrony of the process and the inflammatory response, which would dilute the signal of the myelin genes within the tissue. Consistent with this, we find that, at 6 weeks, a number of the mSCs have been triggered to dedifferentiate as measured by the expression of p75 (Figures S6B and S6C), as a result of the abnormal myelination process.

Moreover, in line with mSCs that lack HDAC3 continuing to remain highly biogenic, mutant mSCs retained the enlarged cytoplasm only seen in control mSCs in their biogenic phase (Figure 7B and quantified in Figure S6D). Moreover, the enlarged cytoplasm was packed with ER, Golgi, and mitochondria and, while larger, resembled normal mSCs in the "factory-like" phase seen only during the post-natal differentiation period (Figures 7C and 6C). Notably, the abnormally high level of myelin production was not associated with increased signaling through the ERK or PI3-kinase pathways (Figure S6E). However, consistent with a highly biogenic state in which the production of proteins is maintained at an abnormally high rate, an ER stress response was triggered in mutant mice but was not detectable in controls (Figure S6F). Notably, the ER-stress response was consistent with the enlarged ER visible in the EM sections at this stage (Figure 7C).

These results indicate that HDAC3 replaces HDAC1/2 at the transition from the biogenic to the homeostatic state and that loss of HDAC3 leads to a prolonged biogenic phase in mutant cells. Consistent with this, in an *in vitro* differentiation assay, we find that, similarly to the process *in vivo*, the transcription rate of the P0 gene drops as differentiation proceeds, (Figure 7D) and this is accompanied by the loss of HDAC2 binding to the P0 enhancer (Figures 7E and S6G). Importantly, HDAC3 continues to bind at the same time point (Figure 1C). Strikingly, *in vivo* analysis showed that, whereas adult mSCs usually express HDAC2 and HDAC1 at very low levels, HDAC2 and HDAC1 levels remained high in adult mSCs lacking HDAC3, consistent with the maintenance of higher levels of myelin gene expression and the continuation of the biogenic state (Figure 7F).

DISCUSSION

A mSC is a highly specialized polarized cell whose function is critical for the normal function of the PNS. The myelin sheath is produced rapidly in the early post-natal period in a remarkable biogenic process. Once formed, the myelin sheath becomes a stable structure, but the components of the sheath still turn over, albeit slowly (Salzer, 2015). In a non-biased screen, we identified HDAC3 as a positive regulator of myelin gene expression. The association of HDAC3 with the activation of gene

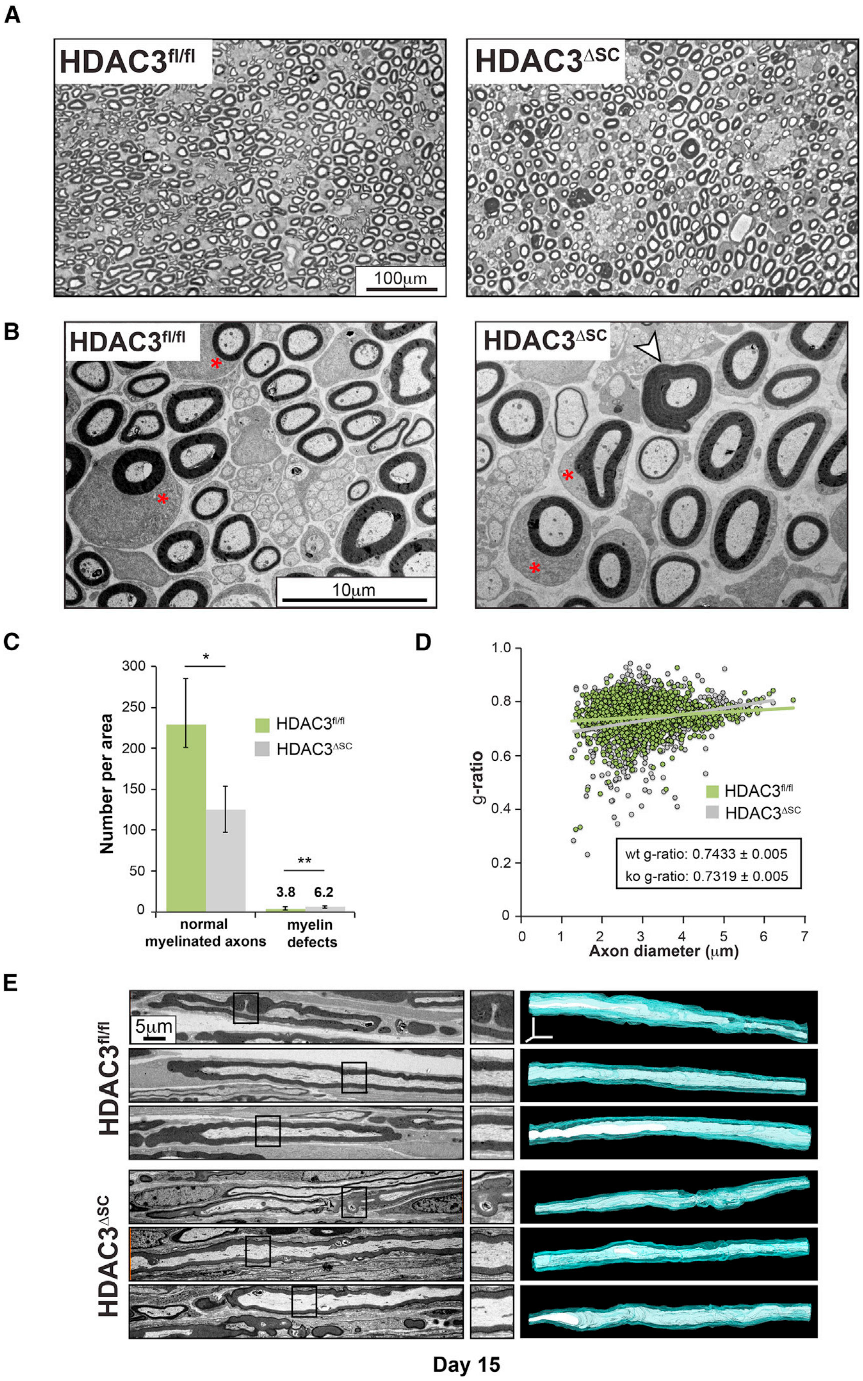

Figure 5. Myelination Defects Become Apparent in Schwann Cells Lacking HDAC3 as Myelination Reaches Completion

(A and B) Representative low- (A) and high- (B) magnification EM images of sciatic nerve sections from postnatal day 15 control (HDAC3$^{fl/fl}$) and mutant (HDAC3$^{\Delta SC}$) mice. Note, while mostly normal, a low percentage of axons show hypermyelination (white arrowhead). In addition, both control and mutant animals show mSCs with an enlarged cytoplasm (red *) indicating that myelination is not complete.

(C) Quantification of normal myelination and the number of axons with myelination defects in HDAC3$^{fl/fl}$ and HDAC3$^{\Delta SC}$ mice (n = 3 mean ± SEM).

(D) Graph shows the g-ratio as a function of axon diameter of the sciatic nerves from postnatal day 15 HDAC3$^{fl/fl}$ and HDAC3$^{\Delta SC}$ mice (n = 3 > 600 axons/genotype).

(E) Representative EM images of longitudinal ultrathin sections (left panel), higher magnification (middle panel), and 3D reconstructions displaying axons (white) and their myelin sheath (blue) (right panel) of sciatic nerves from postnatal day 15 control (HDAC3$^{fl/fl}$) and mutant (HDAC3$^{\Delta SC}$) mice showing, in both cases, one mSC with myelin outfoldings (top) and 2 normal mSCs (bottom).

*$p < 0.05$, **$p < 0.01$. See also Video S2.

expression is consistent with recent studies, particularly those involving oligodendrocyte lineage commitment and neuronal function in which HDAC3 is not acting solely via its more established role of decreasing histone acetylation but rather through the modification and activation of transcriptional complexes (Nott et al., 2016; Zhang et al., 2016). Remarkably, our analysis of HDAC3 function *in vivo* has shown that HDAC3 has a specific role in regulating the transition to a stable myelinating state, when myelin genes need to remain expressed but at lower levels. Failure to transit to this state is associated initially with hypertrophy, polarization abnormalities, and the overproduction of myelin. As myelination is not a synchronous process, the phenotype similarly progressively develops as increasing numbers of mSCs fail to enter the homeostatic state. This then proceeds to severe myelination dysregulation with resultant stress responses, followed finally by axonal loss and the development of severe neuropathies. Importantly, these phenotypes are reminiscent of known human neuropathies, which can be caused by the overproduction of specific myelin genes (Nave et al., 2007).

Our findings have similarities but are clearly distinct from a recent study, which also reported that HDAC3 loss or inhibition can lead to hypermyelination (He et al., 2018). In contrast to our findings, however, they reported that HDAC3 was a negative regulator of myelin gene expression and also proposed HDAC3 inhibition as a mechanism to improve nerve regeneration. Our results would contradict this suggestion. First, we find that loss of HDAC3 does not result in premature myelination but rather normal myelination followed by an ‘‘overshoot’’ with a failure to enter the homeostatic state. This results in grossly aberrant myelination and eventually a severe neuropathy, which is unlikely to be beneficial to the patient.

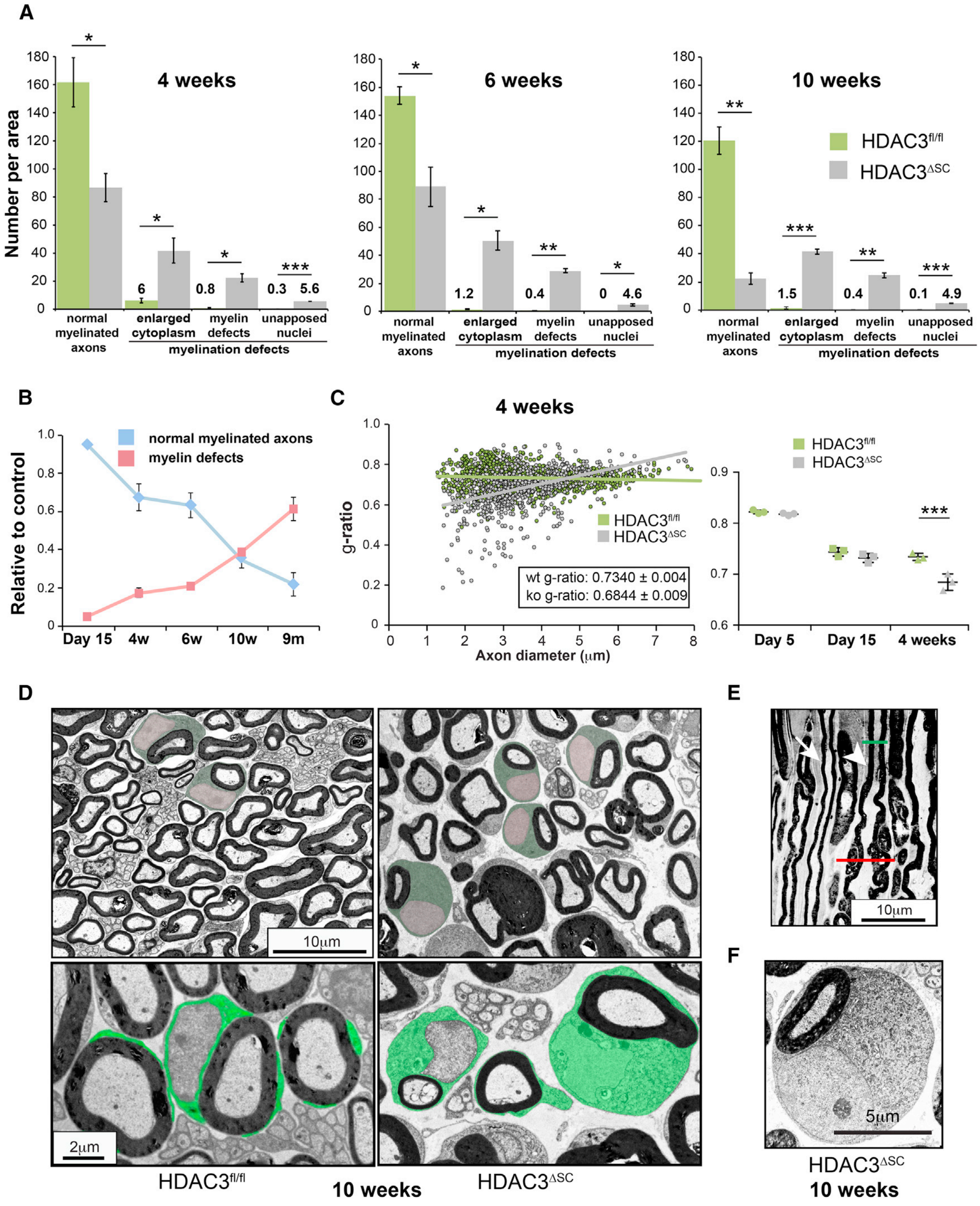

Figure 6. Schwann Cells Lacking HDAC3 Fail to Enter the Homeostatic State

(A) Quantification of myelination defects in the sciatic nerves of HDAC3$^{fl/fl}$ and HDAC3$^{\Delta SC}$ mice at the indicated times (n = 3 mean ± SEM). Note that the graphs in the top panel show that the density of normal mSCs in control animals decreases as the animal ages due to an increase in the overall size of the nerve with age.

(B) Graph shows the accumulation of myelin defects with age observable by normalizing to control levels (n = 3 mean ± SEM).

(C) Graphs show the g-ratio as a function of axon diameter of sciatic nerves from HDAC3$^{fl/fl}$ and HDAC3$^{\Delta SC}$ 4-week-old mice (n = 3 > 600 axons/genotype) (left panel) and the average g-ratio of postnatal day 5, day 15, and 4-week-old HDAC3$^{fl/fl}$ and HDAC3$^{\Delta SC}$ mice (n = 3 mean ± SEM).

(D) Representative colored EM images of control (HDAC3$^{fl/fl}$) and mutant (HDAC3$^{\Delta SC}$) mice. The cytoplasm is colored green with nuclei colored pink. Note the nuclei remain unapposed to the axon in the mutant mice.

(E) Representative EM image of a longitudinal ultrathin sections of a sciatic nerve from 10-week-old mutant HDAC3$^{\Delta SC}$ animals showing one normal (arrow) and one abnormal (arrowhead) mSC displaying regions where the myelin sheath looks normal (green line) and some with outfoldings of myelin (red line).

(F) High-magnification EM image showing the enlarged cytoplasm and unapposed nuclei in the adult mutant mouse (10-week-old mouse).

*p < 0.05, **p < 0.01, ***p < 0.001. See also Figure S5.

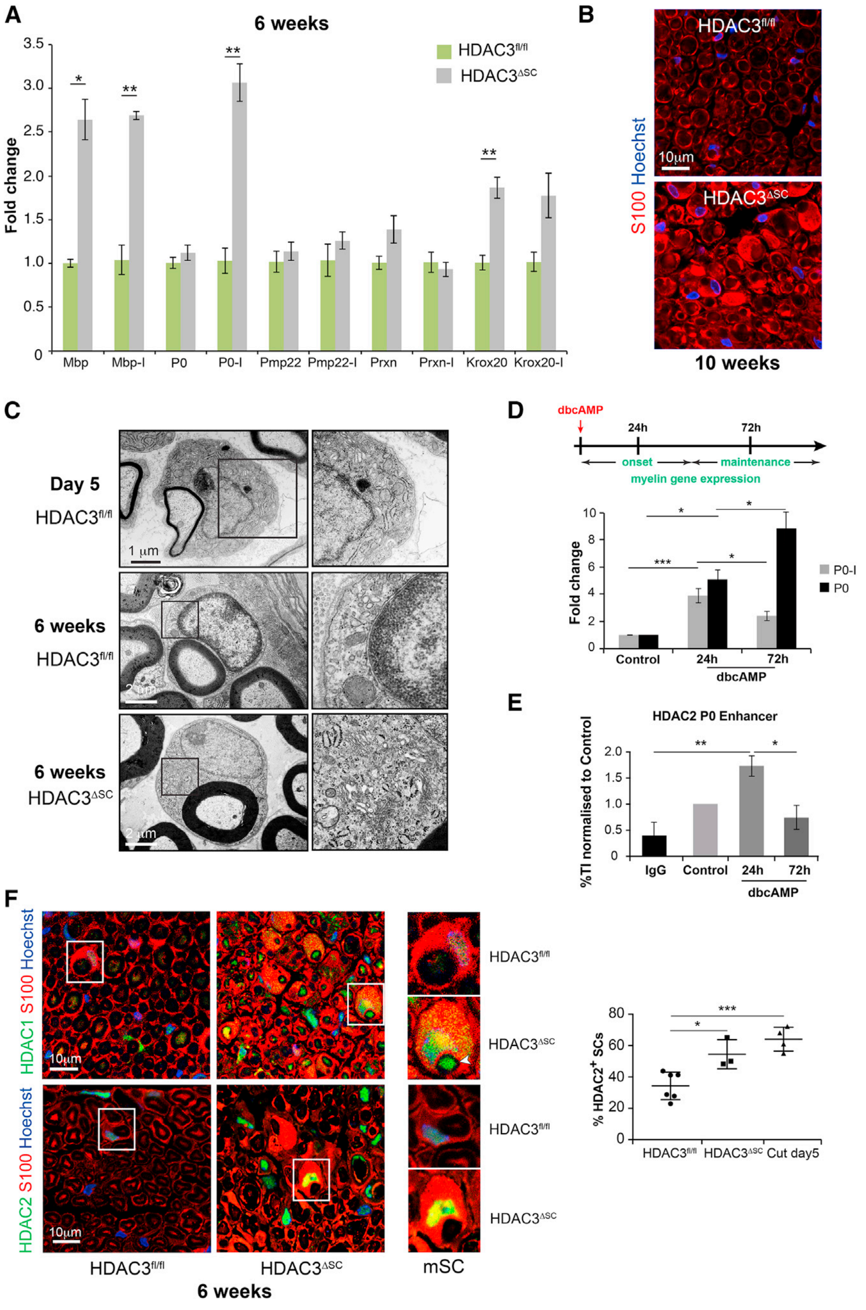

Figure 7. mSCs Lacking HDAC3 Remain in the Biogenic State

(A) RT-qPCR analysis of key myelin genes and the Krox-20 transcription factor at 6 weeks of age. Primers that detect total mRNA or specific primer pairs (-I) that detect only nascent pre-mRNA were used. Prxn, periaxin (n = 4, mean ± SEM).

(B) S100 staining to detect the cytoplasm of SCs shows the enlarged cytoplasm of the mSCs lacking HDAC3. Nuclei are labeled with Hoechst (blue).

(C) Representative EM images showing that the enlarged cytoplasm of mSCs lacking HDAC3 (HDAC3$^{\Delta SC}$) is packed with organelles such as rough endoplasmic reticulum (RER) and mitochondria as seen in normal mSCS during their most biogenic phase (day 5).

(D) RT-qPCR analysis of P0 mRNA (P0) and pre-mRNA (P0-I) of SCs differentiated by the addition of 1mM dbcAMP for the indicated times (n = 4 mean ± SEM).

(E) Graph shows ChIP analysis to detect HDAC2 bound to the P0 enhancer in SCs differentiated by the addition of 1 mM dbcAMP for the indicated times (n = 4 mean ± SEM).

(F) Representative immunofluorescence images showing the expression of HDAC1 and HDAC2 in transverse sections of sciatic nerves of 6-week-old control (HDAC3$^{fl/fl}$) and mutant (HDAC3$^{\Delta SC}$) mice. Graph shows the average percentage of HDAC2^{+} mSCs in sciatic nerves from control (HDAC3$^{fl/fl}$) and mutant (HDAC3$^{\Delta SC}$) mice and in injured sciatic nerves (mean ± SEM). Each dot represents an individual animal.

*$p < 0.05$, **$p < 0.01$, ***$p < 0.001$. See also Figure S6.

Moreover, following a transection injury, the major issue is not the rate of remyelination but rather the speed of axonal regrowth and a failure of axons to regrow back to their original targets because of the disruption to the conduits following the transection (Nguyen et al., 2002). It is thus highly unlikely that a treatment that promotes hypermyelination would improve this situation. Mechanistically, our findings are also distinct. We found that while HDAC1 and 2 are associated with the biogenic/developmental phase, HDAC3 controls the entry to the adult homeostatic state. Consistent with this hypothesis, we also find that, following injury, HDAC1/2 and HDAC3 show different expression patterns when remyelination is required, in that HDAC1/2 expression is re-induced, whereas HDAC3 nuclear expression clearly decreases. This switch from the biogenic to the homeostatic state is associated with the replacement of HDAC2 by HDAC3 on the transcriptional regulatory elements of myelin genes leading to lower levels of myelin gene expression. Loss of HDAC3 results in a failure to exit the biogenic phase and, consistent with this, HDAC1/2 remains expressed into adulthood with a resulting failure to enter the homeostatic state and the development of the hypermyelination phenotype.

The transition to a homeostatic state is a property of many differentiated cells, particularly post-mitotic cells including neurons, muscle cells, endothelial cells, and other types of glia. When cells differentiate it usually involves the relatively rapid production of material new and specific to this new cell state. However, once the transition is complete, many of these cells aim to remain more or less the same throughout adulthood (Lloyd, 2013). This requires a stable, usually lower, level of transcription of many of the same genes induced at high rates during

differentiation. This state can change in a regenerative cell such as a SC or a peripheral neuron, when upon injury the regenerative process will involve the reinitiation of a more biogenic phase (Cattin and Lloyd, 2016; Ma and Willis, 2015). In contrast, in pathological situations, abnormal overproduction by a cell is associated with hypertrophy (such as cardiac hypertrophy), degenerative disorders such as CMT disease, developmental brain disorders, and cancer (Crino, 2011; Lloyd, 2013). While the events controlling switches in differentiation state have been heavily studied, the less dramatic but critically important transition to a homeostatic state is still poorly understood. It is likely to involve many mechanisms, but here we propose the differential use of HDACs as one key mechanism governing the switch between the onset and the maintenance of the myelinating state. Further studies will determine the full mechanistic implications of this switch between HDAC2 and HDAC3. However, our findings offer a unique insight into how these important transitions can be achieved and are likely to have parallels in many similar cell state transitions.

STAR★METHODS

Detailed methods are provided in the online version of this paper and include the following:

- KEY RESOURCES TABLE
- CONTACT FOR REAGENT AND RESOURCE SHARING
- EXPERIMENTAL MODEL AND SUBJECT DETAILS
  - Generation of Schwann cell-specific mutant mice
  - Mouse husbandry
  - Schwann cell culture and NSΔRafER cell generation and culture
- METHOD DETAILS
  - siRNA knockdown
  - Dual luciferase assay
  - Sciatic nerve protein analysis
  - Western Blotting
  - qRT-PCR
  - Immunofluorescence
  - Nerve histology, histomorphometry, electron microscopy and g-ratio calculations
  - 3D reconstruction of myelinated axons
  - ChIP
  - Behavioral Studies
- QUANTIFICATION AND STATISTICAL ANALYSIS

SUPPLEMENTAL INFORMATION

Supplemental Information includes six figures, two tables, and two videos and can be found with this article online at https://doi.org/10.1016/j.celrep.2018.11.045.

ACKNOWLEDGMENTS

This work was supported mainly by a programme grant from Cancer Research UK (C378/A4308) and core support by MRC funding to the MRC LMCB University Unit at UCL, award code MC_U12266B. We would like to thank UCL Biological Services, Julian Hoving and Liza Malong, and the rest of the Lloyd lab for useful discussions.

AUTHOR CONTRIBUTIONS

A.C.L. and L.H.R. conceived the project and together with A.C. wrote the manuscript. L.H.R., A.C., X.F., and A.C.L. designed the experiments. J.J.B. and I.J.W. performed the EM analysis, L.H.R., A.C., A.C.L., X.F., E.H.-W., and C.P. performed the majority of the experiments. I.N. and V.Q. performed some mice studies, L.H.R., J.F. and R.K. performed the initial screen, and J.G.S. and A.R. performed and helped with the ChIP analysis.

DECLARATION OF INTERESTS

The authors declare no competing interests.

Received: February 26, 2018
Revised: October 16, 2018
Accepted: November 9, 2018
Published: December 4, 2018

REFERENCES

Adlkofer, K., Frei, R., Neuberg, D.H., Zielasek, J., Toyka, K.V., and Suter, U. (1997). Heterozygous peripheral myelin protein 22-deficient mice are affected by a progressive demyelinating tomaculous neuropathy. J. Neurosci. *17*, 4662–4671.

Bolis, A., Coviello, S., Bussini, S., Dina, G., Pardini, C., Previtali, S.C., Malaguti, M., Morana, P., Del Carro, U., Feltri, M.L., et al. (2005). Loss of Mtmr2 phosphatase in Schwann cells but not in motor neurons causes Charcot-Marie-Tooth type 4B1 neuropathy with myelin outfoldings. J. Neurosci. *25*, 8567–8577.

Brügger, V., Engler, S., Pereira, J.A., Ruff, S., Horn, M., Welzl, H., Münger, E., Vaquié, A., Sidiropoulos, P.N., Egger, B., et al. (2015). HDAC1/2-dependent P0 expression maintains paranodal and nodal integrity independently of myelin stability through interactions with neurofascins. PLoS Biol. *13*, e1002258.

Brügger, V., Duman, M., Bochud, M., Münger, E., Heller, M., Ruff, S., and Jacob, C. (2017). Delaying histone deacetylase response to injury accelerates conversion into repair Schwann cells and nerve regeneration. Nat. Commun. *8*, 14272.

Camargo, N., Smit, A.B., and Verheijen, M.H.G. (2009). SREBPs: SREBP function in glia-neuron interactions. FEBS J. *276*, 628–636.

Cardona, A., Saalfeld, S., Preibisch, S., Schmid, B., Cheng, A., Pulokas, J., Tomancak, P., and Hartenstein, V. (2010). An integrated micro- and macroarchitectural analysis of the Drosophila brain by computer-assisted serial section electron microscopy. PLoS Biol. *8*, e1000502.

Cattin, A.L., and Lloyd, A.C. (2016). The multicellular complexity of peripheral nerve regeneration. Curr. Opin. Neurobiol. *39*, 38–46.

Crino, P.B. (2011). mTOR: A pathogenic signaling pathway in developmental brain malformations. Trends Mol. Med. *17*, 734–742.

D'Antonio, M., Musner, N., Scapin, C., Ungaro, D., Del Carro, U., Ron, D., Feltri, M.L., and Wrabetz, L. (2013). Resetting translational homeostasis restores myelination in Charcot-Marie-Tooth disease type 1B mice. J. Exp. Med. *210*, 821–838.

Decker, L., Desmarquet-Trin-Dinh, C., Taillebourg, E., Ghislain, J., Vallat, J.M., and Charnay, P. (2006). Peripheral myelin maintenance is a dynamic process requiring constant Krox20 expression. J. Neurosci. *26*, 9771–9779.

Farley, F.W., Soriano, P., Steffen, L.S., and Dymecki, S.M. (2000). Widespread recombinase expression using FLPeR (flipper) mice. Genesis *28*, 106–110.

Feltri, M.L., D'Antonio, M., Previtali, S., Fasolini, M., Messing, A., and Wrabetz, L. (1999). P0-Cre transgenic mice for inactivation of adhesion molecules in Schwann cells. Ann. N.Y. Acad. Sci. *883*, 116–123.

Feltri, M.L., Graus Porta, D., Previtali, S.C., Nodari, A., Migliavacca, B., Cassetti, A., Littlewood-Evans, A., Reichardt, L.F., Messing, A., Quattrini, A., et al. (2002). Conditional disruption of beta 1 integrin in Schwann cells impedes interactions with axons. J. Cell Biol. *156*, 199–209.

Garbay, B., Heape, A.M., Sargueil, F., and Cassagne, C. (2000). Myelin synthesis in the peripheral nervous system. Prog. Neurobiol. *61*, 267–304.

Haberland, M., Montgomery, R.L., and Olson, E.N. (2009). The many roles of histone deacetylases in development and physiology: Implications for disease and therapy. Nat. Rev. Genet. *10*, 32–42.

Harrisingh, M.C., Perez-Nadales, E., Parkinson, D.B., Malcolm, D.S., Mudge, A.W., and Lloyd, A.C. (2004). The Ras/Raf/ERK signalling pathway drives Schwann cell dedifferentiation. EMBO J. *23*, 3061–3071.

He, X., Zhang, L., Queme, L.F., Liu, X., Lu, A., Waclaw, R.R., Dong, X., Zhou, W., Kidd, G., Yoon, S.O., et al. (2018). A histone deacetylase 3-dependent pathway delimits peripheral myelin growth and functional regeneration. Nat. Med. *24*, 338–351.

Hung, H., Kohnken, R., and Svaren, J. (2012). The nucleosome remodeling and deacetylase chromatin remodeling (NuRD) complex is required for peripheral nerve myelination. J. Neurosci. *32*, 1517–1527.

Jacob, C. (2017). Chromatin-remodeling enzymes in control of Schwann cell development, maintenance and plasticity. Curr. Opin. Neurobiol. *47*, 24–30.

Jacob, C., Christen, C.N., Pereira, J.A., Somandin, C., Baggiolini, A., Lötscher, P., Ozçelik, M., Tricaud, N., Meijer, D., Yamaguchi, T., et al. (2011). HDAC1 and HDAC2 control the transcriptional program of myelination and the survival of Schwann cells. Nat. Neurosci. *14*, 429–436.

Jessen, K.R., and Mirsky, R. (2005). The origin and development of glial cells in peripheral nerves. Nat. Rev. Neurosci. *6*, 671–682.

LeBlanc, S.E., Jang, S.W., Ward, R.M., Wrabetz, L., and Svaren, J. (2006). Direct regulation of myelin protein zero expression by the Egr2 transactivator. J. Biol. Chem. *281*, 5453–5460.

Lin, S.H., Cheng, Y.R., Banks, R.W., Min, M.Y., Bewick, G.S., and Chen, C.C. (2016). Evidence for the involvement of ASIC3 in sensory mechanotransduction in proprioceptors. Nat. Commun. *7*, 11460.

Lloyd, A.C., Obermuller, F., Staddon, S., Barth, C.F., McMahon, M., and Land, H. (1997). Cooperating oncogenes converge to regulate cyclin/cdk complexes. Gene Dev. *11*, 663–677.

Lloyd, A.C. (2013). The regulation of cell size. Cell *154*, 1194–1205.

Ma, T.C., and Willis, D.E. (2015). What makes a RAG regeneration associated? Front. Mol. Neurosci. *8*, 43.

Malik, A.N., Vierbuchen, T., Hemberg, M., Rubin, A.A., Ling, E., Couch, C.H., Stroud, H., Spiegel, I., Farh, K.K., Harmin, D.A., and Greenberg, M.E. (2014). Genome-wide identification and characterization of functional neuronal activity-dependent enhancers. Nat. Neurosci. *17*, 1330–1339.

Mallon, B.S., Shick, H.E., Kidd, G.J., and Macklin, W.B. (2002). Proteolipid promoter activity distinguishes two populations of NG2-positive cells throughout neonatal cortical development. J. Neurosci. *22*, 876–885.

Mitchell, P.J., Perez-Nadales, E., Malcolm, D.S., and Lloyd, A.C. (2003). Dissecting the contribution of p16(INK4A) and the Rb family to the Ras transformed phenotype. Mol. Cell Biol. *23*, 2530–2542.

Monk, K.R., Feltri, M.L., and Taveggia, C. (2015). New insights on Schwann cell development. Glia *63*, 1376–1393.

Montgomery, R.L., Potthoff, M.J., Haberland, M., Qi, X.X., Matsuzaki, S., Humphries, K.M., Richardson, J.A., Bassel-Duby, R., and Olson, E.N. (2008). Maintenance of cardiac energy metabolism by histone deacetylase 3 in mice. J. Clin. Invest. *118*, 3588–3597.

Nave, K.A. (2010). Myelination and the trophic support of long axons. Nat. Rev. Neurosci. *11*, 275–283.

Nave, K.A., and Werner, H.B. (2014). Myelination of the nervous system: Mechanisms and functions. Annu. Rev. Cell Dev. Biol. *30*, 503–533.

Nave, K.A., Sereda, M.W., and Ehrenreich, H. (2007). Mechanisms of disease: Inherited demyelinating neuropathies–from basic to clinical research. Nat. Clin. Pract. Neurol. *3*, 453–464.

Nguyen, Q.T., Sanes, J.R., and Lichtman, J.W. (2002). Pre-existing pathways promote precise projection patterns. Nat. Neurosci. *5*, 861–867.

Nott, A., Cheng, J., Gao, F., Lin, Y.T., Gjoneska, E., Ko, T., Minhas, P., Zamudio, A.V., Meng, J., Zhang, F., et al. (2016). Histone deacetylase 3 associates with MeCP2 to regulate FOXO and social behavior. Nat. Neurosci. *19*, 1497–1505.

Parrinello, S., and Lloyd, A.C. (2009). Neurofibroma development in NF1—insights into tumour initiation. Trends Cell Biol. *19*, 395–403.

Pereira, J.A., Lebrun-Julien, F., and Suter, U. (2012). Molecular mechanisms regulating myelination in the peripheral nervous system. Trends Neurosci. *35*, 123–134.

Quintes, S., Brinkmann, B.G., Ebert, M., Fröb, F., Kungl, T., Arlt, F.A., Tarabykin, V., Huylebroeck, D., Meijer, D., Suter, U., et al. (2016). Zeb2 is essential for Schwann cell differentiation, myelination and nerve repair. Nat. Neurosci. *19*, 1050–1059.

Roberts, S.A., and Lloyd, A.C. (2012). Aspects of cell growth control illustrated by the Schwann cell. Curr. Opin. Cell Biol. *24*, 852–857.

Salzer, J.L. (2015). Schwann cell myelination. Cold Spring Harb. Perspect. Biol. Published online June 8, 2015. https://doi.org/10.1101/cshperspect.a020529.

Schindelin, J., Arganda-Carreras, I., Frise, E., Kaynig, V., Longair, M., Pietzsch, T., Preibisch, S., Rueden, C., Saalfeld, S., Schmid, B., et al. (2012). Fiji: An open-source platform for biological-image analysis. Nat. Methods *9*, 676–682.

Seto, E., and Yoshida, M. (2014). Erasers of histone acetylation: The histone deacetylase enzymes. Cold Spring Harb. Perspect. Biol. *6*, a018713.

Stalling, D., Westerhoff, M., and Hege, H.-C. (2005). Amira: A highly interactive system for visual data analysis. In The Visualization Handbook, C.D. Hansen and C.R. Johnson, eds. (Academic Press), pp. 749–767.

Stolt, C.C., and Wegner, M. (2016). Schwann cells and their transcriptional network: Evolution of key regulators of peripheral myelination. Brain Res. *1641* (*Pt A*), 101–110.

Tersar, K., Boentert, M., Berger, P., Bonneick, S., Wessig, C., Toyka, K.V., Young, P., and Suter, U. (2007). Mtmr13/Sbf2-deficient mice: An animal model for CMT4B2. Hum. Mol. Genet. *16*, 2991–3001.

Topilko, P., Schneider-Maunoury, S., Levi, G., Baron-Van Evercooren, A., Chennoufi, A.B., Seitanidou, T., Babinet, C., and Charnay, P. (1994). Krox-20 controls myelination in the peripheral nervous system. Nature *371*, 796–799.

Toyama, B.H., Savas, J.N., Park, S.K., Harris, M.S., Ingolia, N.T., Yates, J.R., 3rd, and Hetzer, M.W. (2013). Identification of long-lived proteins reveals exceptional stability of essential cellular structures. Cell *154*, 971–982.

Vallat, J.M., Mathis, S., and Funalot, B. (2013). The various Charcot-Marie-Tooth diseases. Curr. Opin. Neurol. *26*, 473–480.

Wang, Z., Zang, C., Cui, K., Schones, D.E., Barski, A., Peng, W., and Zhao, K. (2009). Genome-wide mapping of HATs and HDACs reveals distinct functions in active and inactive genes. Cell *138*, 1019–1031.

Wu, L.M., Wang, J., Conidi, A., Zhao, C., Wang, H., Ford, Z., Zhang, L., Zweier, C., Ayee, B.G., Maurel, P., et al. (2016). Zeb2 recruits HDAC-NuRD to inhibit Notch and controls Schwann cell differentiation and remyelination. Nat. Neurosci. *19*, 1060–1072.

Zhang, L., He, X., Liu, L., Jiang, M., Zhao, C., Wang, H., He, D., Zheng, T., Zhou, X., Hassan, A., et al. (2016). Hdac3 interaction with p300 histone acetyltransferase regulates the oligodendrocyte and astrocyte lineage fate switch. Dev. Cell *36*, 316–330.

Zupkovitz, G., Tischler, J., Posch, M., Sadzak, I., Ramsauer, K., Egger, G., Grausenburger, R., Schweifer, N., Chiocca, S., Decker, T., and Seiser, C. (2006). Negative and positive regulation of gene expression by mouse histone deacetylase 1. Mol. Cell. Biol. *26*, 7913–7928.

STAR★METHODS

KEY RESOURCES TABLE

REAGENT or RESOURCE	SOURCE	IDENTIFIER
Antibodies		
Rabbit monoclonal anti-HDAC2 (clone Y461) IF: 1/400	Abcam	Cat#ab32117 RRID:AB_732777
Mouse monoclonal anti-HDAC2 ChIP and WB: 1/1000	Abcam	Cat#ab12169 RRID:AB_2118547
Rabbit polyclonal anti-HDAC3 ChIP, WB: 1/1000 and IF: 1/400	Abcam	Cat#ab7030 RRID:AB_305708
Rabbit polyclonal anti-HDAC1 IF: 1/200	Abcam	Cat#ab7028 RRID:AB_305705
Chicken polyclonal anti- 200kD neurofilament IF: 1/1000	Abcam	Cat# ab4680 RRID: AB_30456
Rabbit polyclonal S100 IF: 1/1000	Dako	Cat#Z0311 RRID:AB_10013383
Rat anti-mouse CD31 platelet endothelial cell adhesion molecule (PECAM) IF: 1/50	BD Biosciences	Cat# 553370 RRID: AB_394816
Mouse monoclonal S100 (clone SH-B1) IF: 1/500	Sigma-Aldrich	Cat#S2532 RRID: AB_477499
Rabbit polyclonal anti-nerve growth factor (NGF-receptor) p75 IF: 1/400	Millipore	Cat# AB1554 RRID: AB_90760
Rat F4/80 monoclonal (clone Cl:A3-1) IF: 1/100	Bio-Rad	Cat#MCA497 RRID:AB_2098196
Rabbit polyclonal ERK WB: 1/1000	Sigma-Aldrich	Cat#M5670 RRID: AB_477216
Rabbit monoclonal P-Akt(Thr308) (clone C31E5E) WB: 1/1000	Cell Signaling Technology	Cat#2965S RRID: AB_2255933
Rabbit monoclonal P-Akt(Ser473) (clone 193H12) WB: 1/1000	Cell Signaling Technology	Cat#2336S RRID:AB_491022
Rabbit polyclonal P-ERK WB: 1/1000	Cell Signaling Technology	Cat#4370S RRID: 2315112
Chicken polyclonal anti-myelin protein zero (P0) WB: 1/1000	Abcam	Cat#ab39375 RRID:AB_881430
Rabbit polyclonal anti-GRP78 BiP WB: 1/1000	Abcam	Cat# ab21685 RRID:AB_2119834
Goat anti-mouse Alexa Fluor 488 (IF)	Thermo Fisher Scientific	Cat#A11001 RRID:AB_2534069
Goat anti-rat Alexa Fluor 488 (IF)	Thermo Fisher Scientific	Cat#A11006 RRID:AB_2534074
Goat anti-Rabbit Alexa Fluor 594 (IF)	Thermo Fisher Scientific	Cat#A11012 RRID:AB_2534079
Goat anti-Mouse Alexa Fluor 647 (IF)	Thermo Fisher Scientific	Cat#21235 RRID:AB_141693
Goat anti-Chichen Alexa Fluor 647 (IF)	Thermo Fisher Scientific	Cat#A21449 RRID:AB_2535866
Sheep anti-mouse IgG, HRP	GE Healthcare	Cat#NA931 RRID:AB_772210
Donkey anti-rabbit IgG, HRP	GE Healthcare	Cat#NA934 RRID:AB_772206
Donkey anti-chicken IgG, HRP	Sigma-Aldrich	Cat#AP194P, RRID:AB_92682
Bacterial and Virus Strains		
NSΔRafER retrovirus	Lloyd et al., 1997	N/A
Chemicals, Peptides, and Recombinant Proteins		
4-hydroxytamoxifen	Sigma-Aldrich	Cat#H7904
dbcAMP	Sigma-Aldrich	Cat#DO627
insulin	Sigma-Aldrich	Cat#19278
hiperfect	QIAGEN	Cat#301707
attractene	QIAGEN	Cat#301005
Critical Commercial Assays		
PureLink RNA Micro kit	Thermo Fischer Scientific	Cat#12183016
Dual Luciferase Reporter assay kit	Promega	Cat#E1910
Deposited Data		
Raw and analyzed data	This paper	N/A
Experimental Models: Organisms/Strains		
Crl:CD(SD) Rattus norvegicus	Charles River	Cat#734476, RRID:RGD_734476
C57BL/6N- Hdac3$^{tm1a(EUCOMM)Wtsi}$ *Mus musculus*	EMMA	Cat# WTSI:3856 RRID:IMSR_ WTSI:3856

(*Continued on next page*)

Continued

REAGENT or RESOURCE	SOURCE	IDENTIFIER
Tg(Mpz-cre)26Mes *Mus musculus*	Feltri et al., 1999	N/A
Mouse: PLP-eGFP	Mallon et al., 2002	N/A
Oligonucleotides		
Primer for genotyping: HDAC3 Forward: ACCATGTGTCAAAGGAA CAGTG	This paper	N/A
Primer for genotyping: HDAC3 Reverse WT allele: GGTAACAACTG CCATGGAAACA	This paper	N/A
Primer for genotyping: HDAC3 Reverse targeted allele: GGGAAAGG GTTCGAAGTTTCCTA	This paper	N/A
Primer for genotyping: P0-Cre 1: CGGTCGATGCAACGAGTGATGAG	This paper	N/A
Primer for genotyping: P0-Cre 2: CCAGAGACGGAAATCCATCGCTC	This paper	N/A
Primers for RT-qPCR, see Table S1	This paper	N/A
Primers for ChIP, see Table S2	This paper	N/A
Si RNA targeting sequence: HDAC3 siRNA1: GAACUUCCCUAUAG UGAAU	Dharmacon	Cat#D-093064-01-0005
Si RNA targeting sequence: HDAC3 siRNA2: CGCCUGGCAUUGA CUCAUA	Dharmacon	Cat#D-093064-04-0005
Recombinant DNA		
P0 promoter enhancer construct: pGL3-P0-Int-Pro	LeBlanc et al., 2006	N/A
Renilla construct: pRL-CMV	Promega	Cat#E2261
Software and Algorithms		
Fiji	Schindelin et al., 2012	https://imagej.net/Fiji/Downloads
Amira	Stalling et al., 2005	https://www.fei.com/software/amira-for-cell-biology/
Prism	GraphPad	https://www.graphpad.com/scientific-software/prism/

CONTACT FOR REAGENT AND RESOURCE SHARING

Further information and requests for resources and reagents should be directed to and will be fulfilled by the Lead Contact, Alison Lloyd (alison.lloyd@ucl.ac.uk).

EXPERIMENTAL MODEL AND SUBJECT DETAILS

Generation of Schwann cell-specific mutant mice

HDAC3 mutant mice were generated using mice heterozygous for the Hdac3tm1a(EUCOMM)Wtsi allele carrying a neomycin (Neo) resistance gene flanked by two FRT sites and two loxP sites that flank exon 3 from the European Mouse Mutant Archive (EMMA, http://www.emma.org). Hdac3tm1a(EUCOMM)Wtsi /+ mice were crossed with FLP deleted mice (Farley et al., 2000) to remove the Neo cassette (generous gift from Josef Kittler), leaving the Hdac3 exon 3 floxed by a pair of loxP site. Deletion of the floxed region was achieved specifically in SCs by further crossing to P0-Cre mice (Feltri et al., 1999). Genotyping was performed by PCR of genomic DNA using the following primers:

HDAC3 wt allele: ACCATGTGTCAAAGGAACAGTG and GGTAACAACTGCCATGGAAACA
HDAC3 targeted allele: ACCATGTGTCAAAGGAACAGTG
and GGGAAAGGGTTCGAAGTTTCCTA
P0-Cre 1: CGGTCGATGCAACGAGTGATGAGG
P0-Cre 2: CCAGAGACGGAAATCCATCGCTCG

Crosses with PLP-EGFP transgenic mice (Mallon et al., 2002) were performed for studies requiring GFP+ SCs in control (HDAC3$^{fl/fl}$) and mutant mice (HDAC3$^{\Delta SC}$).

Mouse husbandry

Mice were group-housed in a 12 hr light/dark cycle (light between 07:00 and 19:00) in a temperature-controlled room (21.1 ± 1.1°C) with free access to water and food. The ages of mice are indicated in the figure legends or methods. Sex was not determined for neonatal pups. For analyses of older animals (> P15), both males and females were used.

Animal work was carried out in accordance to UK Home Office regulations.

Schwann cell culture and NSΔRafER cell generation and culture

Rat primary SCs were cultured in DMEM- low glucose (1g/L) (Lonza) supplemented with 3% fetal bovine serum (FBS, Labtech.com) 1 μM forskolin (Abcam), 200nM L-Glutamine (GIBCO), Neuregulin, 100 μg/ml kanamycin and 800 μg/ml gentamycin (GIBCO) on poly-L-lysine coated tissue culture plates and maintained at 37°C and 10% CO_2.

SCs were infected by co-cultivation at a 1:2 ratio with a ΔRafER expressing producer line that had been pre-treated with mitomycin C (Lloyd et al., 1997). After two to three days, cultures were transferred to selective media containing 400 μg/ml G418 (GIBCO) and the resulting drug resistant colonies pooled and expanded.

For the differentiation assays, NSΔRafER cells were washed and cultured in serum-free SATO defined medium (Mitchell et al., 2003). Cells were then induced to differentiate by the addition of 1mM dbcAMP (Sigma). To induce their dedifferentiation, ΔRafER was activated by the addition of 100nM hydroxytamoxifen (Sigma) in ethanol (Harrisingh et al., 2004).

METHOD DETAILS

siRNA knockdown

siRNA HDAC3 knockdown was performed in NSΔRafER cells using HiPerFect (QIAGEN). siRNA target sequences used at the indicated concentrations were:

SiRNA1: GAACUUCCCUAUAGUGAAU at 5nM
SiRNA2: CGCCUGGCAUUGACUCAUA at 5nM

To test knockdown efficiency, cells were lysed in RIPA buffer (1% triton, 0.5% Na deoxycholate, 1mM EGTA, 50mM Tris pH 7.5, protease and phosphatase inhibitors) and standard Western Blotting was performed.

Dual luciferase assay

To assess the effect of silencing HDAC3 on P0 enhancer-promoter activity, NSΔRafER cells were cultured in 6-well plates until 80% confluence was reached. Cells were transfected with 300ng pGL3-P0-Int-Pro luciferase vector (firefly), 5ng of Renilla and 5nM of scrambled (QIAGEN), HDAC3 #1 or #2 siRNA (Dharmacon) with Attractene (Invitrogen). Complexes were added to the cells and incubated for 2h at 37°C. Cells were lysed 48h following transfection with a passive lysis buffer (Promega). Samples were processed using the dual luciferase reporter assay system (Promega) according to the manufacturer's instructions.

Sciatic nerve protein analysis

Immediately after dissection, sciatic nerves were snap-frozen in liquid nitrogen and stored at −80°C until further processing. To prepare protein lysates, nerves were crushed on dry ice, mixed with lysis buffer (25 mM Tris-HCl pH 7.4, 95 mM NaCl, 10 mM EDTA, 2% SDS, protease and phosphatase inhibitors (Sigma)), boiled for 5 minutes, and spun for 15 minutes. The supernatant of each sample was adjusted to the same concentration and mixed 1:4 with sample buffer (200 mM Tris-HCl pH6.8, 40% glycerol, 8% SDS, 20% β-mercaptoethanol, 0.4% bromophenol blue).

Western Blotting

Western blotting was performed using Hoefer Scientific Instrument apparatus and BioRad Western Blot electrophoresis system. 20-30 μg of protein was resolved using sodium dodecyl sulfate – polyacrylamide gel electrophoresis. Proteins were transferred onto a nitrocellulose membrane (Millipore-Immobilon), which was then blocked in 5% milk-TBST for 1 hour at room temperature (RT) and incubated overnight at 4°C with the relevant primary antibody (see Key Resources Table). The following day, 3 washes in TBS-T were performed before incubation with the appropriate HRP-conjugated secondary antibody (anti-mouse, GE Healthcare NA931; antirabbit, GE Healthcare NA934; anti-chicken, Sigma-Aldrich AP194P) for 1 hour at RT at 1/5000. After 3 additional washes in TBS-T, proteins of interest were detected with Pierce- ECL western blot substrate (Thermo Scientific) or Luminata Crescendo Western HRP substrate (EMD-Millipore) and images acquired on the Imagequant LAS 4000.

qRT-PCR

Immediately after dissection, sciatic nerves were snap-frozen in liquid nitrogen and stored at −80°C until further processing. Nerves were crushed and homogenized on dry ice and then lysed in Trizol Reagent (Ambion). Nerves of three control or mutant animals were pooled for P5 and P15 analysis; nerves of two animals were pooled when the analysis was performed at 6 weeks. Cells were directly lysed in Trizol Reagent. RNA purification was performed using PureLinkTM RNA Mini kit (ThermoFischer Scientific). Manufacturer

instructions were followed with an additional step of DNase treatment (QIAGEN) to degrade the genomic DNA. 500ng-1 μg/μl of RNA was then reverse-transcribed using Super-Script II Reverse Transcriptase (Invitrogen) and quantitative PCR (qPCR) was then performed using the MESA Blue qPCR Kit (Eurogentec). 5 μL of template cDNA and 20 μL of MESA blue qPCR MasterMix Plus kit (Eurogentec) including 100nM forward and reverse primers (see sequences in Table S1) were used per reaction in a 96-well plate. Water was used as a negative control. Relative expression values for each gene of interest were obtained after normalizing to b2m using the primers described in Table S1.

Immunofluorescence

Sciatic nerves were dissected and fixed for a minimum of 4 hours in 4% PFA/PBS, cryoprotected in 30% sucrose/PBS overnight at 4°C, incubated in 50% OCT/30% sucrose/PBS for 2 hours, embedded in OCT and finally frozen in liquid nitrogen. Cross cryosections (12 μm) were cut using a cryostat (Leica), permeabilised in 0.3% triton/PBS for 30 minutes, washed and then blocked in 10% goat serum (Sigma)/PBS for 1 hour at RT. Primary antibodies were diluted in 10% goat serum/PBS at the indicated concentration (see Key Resources Table) and incubated overnight at 4°C. After washing, the appropriate fluorescent secondary antibody (1/400, Alexa fluor®488, 594 from Thermo Fisher Scientific) was used with Hoechst to counterstain the nuclei for 1 hour at RT. Samples were mounted in Fluoromount G (Southern Biotechnology). For HDAC1 immunostaining, nerve sections were first incubated for 5 minutes in 70% EtOH, washed in PBS and then incubated with proteinase K (Roche) 40 μg/ml for 40 s. After washing, the above described protocol of immunostaining was performed. For HDAC3 immunostaining, nerves were directly embedded in OCT and snap frozen in liquid nitrogen. Cryosections were post-fixed in 4% PFA/PBS, permeabilised in 0.3% triton/PBS for 30 minutes and then blocked in 1/50 affiniPure Fab fragment donkey anti-mouse IgG (Jackson Immunoresearch) 10% goat serum/PBS overnight at 4°C. After washing, the above-described protocol of immunostaining was performed.

Nerve histology, histomorphometry, electron microscopy and g-ratio calculations

Sciatic nerves were dissected and fixed in 2% glutaraldehyde in 0.12M phosphate buffer pH 7.4 for up to 3 days at 4°C. They were then post-fixed in 2% osmium tetroxide overnight at 4°C and block stained with 2% uranyl acetate for 45 minutes at 4°C. Nerves were then dehydrated and embedded in epoxy resin. Semi-thin sections were cut using a diamond Histo knife (Diatome) at 0.1 μm, dried and stained with 0.5% toluidine blue in 2% Borax at 75°C for 30 s. Dried sections were mounted with DPX (Sigma) and representative images were acquired using a wide-field microscope (Zeiss Axio Scope.A1). Ultrathin sections of 70nm were cut with a diamond knife, collected onto formvar coated slot grids and stained with lead citrate. Representative images were acquired with a transmission electron microscope (T12 Tecnai Spirit, Thermo-Fischer) using a Morada camera and iTEM software (Olympus SIS).

Quantification of the number of axons, myelin defects, enlarged cytoplasm and unapposed nuclei was performed on at least 5 images per mouse, 3 mice per genotype. Nerves were blinded prior to analysis to avoid unconscious bias. Image fields of view were 76.95x51.24 microns for mice aged 6 and 10 weeks and 50.49x33.62 microns for mice aged 4 weeks. For the graphs, the same area (76.95x51.24 microns) was corrected for across ages. For 5 day old mice 30.45x20.28 micron image fields of view were used to quantify the number of normal axons, axons with myelin defects and individual sorted axons (specified by a single axon, separate from the axonal bundles and associated with a single SC) and the unsorted axons (specified by the total area of an individual axonal bundle). We also used these images to quantify the size distribution of myelinated axons and the g-ratio. Mean axon diameter (with and without myelin) was measured (10 fields, 3 mice per genotype) and binned based on their diameter. g-ratios of individual axons as a measure of myelin thickness were determined by dividing the mean diameter of an axon without myelin by the mean diameter of the same axon with myelin. All measurements were acquired using Photoshop to draw the axons and their associated myelin sheath and Fiji software (Schindelin et al., 2012) to measure their mean diameter.

3D reconstruction of myelinated axons

More than 90 longitudinal, 70nm thick, serial sections were collected on formvar grids and the same region of axons serially imaged manually, as above. Serial section images were aligned using TrakEM2 plugin (Cardona et al., 2010), segmented and rendered 3D using Amira (Thermo Fischer).

ChIP

ChIP experiments were performed using a modified protocol based upon Malik et al., 2014 (Malik et al., 2014). Briefly, 12 million (4x15cm plates) NSΔRafER cells were used per condition for each experiment. Cells were washed once with PBS, and protein-DNA complexes crosslinked by adding 1% PFA/PBS for 10 minutes at RT. Quenching of crosslinking was achieved by adding 125mM glycine for 5 minutes at RT. Cells were rinsed 3 times with cold PBS, then harvested in PBS. Following centrifugation at 2000 g for 10 minutes at 4°C, cell pellets were snap frozen in liquid nitrogen and stored at −80°C until required. Samples were then resuspended and lysed in 1mL of buffer 1 (50mM HEPES-KOH, pH 7.5, 140mM NaCl, 1mM EDTA, pH 8.0, 10% glycerol, 0.5% NP-40, 0.25% Triton X-100 and complete protease inhibitor cocktail, PMSF, phosphatase inhibitors) per 6 million cells and rotated for 10 minutes at 4°C. Nuclei were pelleted by centrifugation at 1000 g for 10 minutes at 4°C, then washed in 1ml of buffer 2 (200mM NaCl, 1mM EDTA, pH 8.0, 0.5mM

EGTA, pH 8.0, 10mM Tris-HCl, pH 8.0, and complete protease inhibitor cocktail, PMSF, phosphatase inhibitors) per 6 million cells and rotated for 10 minutes at 4°C. Nuclei were again pelleted and resuspended in 400 μL buffer 3 (1mM EDTA, pH 8.0, 0.5mM EGTA,

pH 8.0, 10mM Tris-HCl, pH 8.0, and complete protease inhibitor cocktail, PMSF, phosphatase inhibitors) per 6 million cells. Sonication was carried out using Bioruptor Sonication at high power setting. 30 pulses of 30 s each were delivered at this setting, then insoluble materials were removed by centrifugation at 17000 g for 10 minutes at 4°C. An additional 30 pulses of 30 s each were then delivered, resulting in genomic DNA fragments with sizes ranging from 200bp to 1kb. Supernatant was collected and 2% of the chromatin was reverse crosslinked (15 minutes at 95°C in buffer 3 with 300mM NaCL), PCR purified (QIAGEN) and quantified using Qubit (Thermo Fischer). Samples were then equalised in regards to protein amount and preclearing carried out with 50% bead volume of protein A beads (GE Healthcare) prepared for ChIP (1.5g beads swelled O/N with PBS at 4°C, washed 3 times with TE buffer pH 8.0, resuspended in 20ml TE buffer pH 8.0 with 1.4mg salmon sperm DNA, 6mg BSA, 0.05% NaN3). Following preclearing, supernatant was transferred to a new tube and supplemented so that it contains 165mM NaCL, 1% Triton X and 0.1% sodium deoxycholate. 10% of the sample was then saved as input. The remainder was incubated with 5 μg antibody (2.5 μl) or IgG (12.5 μl) overnight at 4°C (HDAC2, Abcam ab12169**;** mouse IgG, Santa Cruz sc-2025), rotating. The next day, samples were incubated with 50 μL bead volume of protein A beads for 2 hours at 4°C. Beads bound by immune complexes were pelleted by centrifugation at 500 g for 1 minute at 4°C, then washed twice with the following buffers: low-salt buffer (0.1% SDS, 1% Triton X-100, 2mM EDTA, 20mM Tris-HCl, pH 8.1, and 150mM NaCl), high-salt buffer (0.1% SDS, 1% Triton X-100, 2mM EDTA, 20mM Tris-HCl, pH 8.1, and 500mM NaCl) and LiCl buffer (0.25M LiCl, 1% NP40, 1% sodium deoxycholate, 1mM EDTA and 10mM Tris, pH 8.1). For each wash, beads were rotated for 10 minutes at 4°C then spun at 500 g for 1 minute at 4°C. Following the last wash, beads were washed twice with TE buffer. Immunoprecipitated material was eluted by adding 100 μL of elution buffer (10mM Tris-HCl, pH 8.0, 1mM EDTA, pH 8.0, and 1% SDS) to each ChIP reaction, incubating at 65°C 5 minutes with gentle mixing, then incubating at RT with agitation for 10 minutes. Samples were spun at 10,000 g for 10 minutes at RT, supernatant saved, then beads eluted once more. Elution buffer was added to each input so that input volume equals 200 μl. To ChIP samples and inputs, NaCl was added to a final concentration of 300mM and crosslinking was reversed overnight at 65°C. Samples were then purified using the QIAquick PCR purification kit (QIAGEN), and DNA fragments were eluted in 40 μL of buffer EB/3 (elution buffer consisting of 10mM Tris-HCl, pH 8.5; QIAGEN, diluted in water). Samples were assessed by gel or qPCR using primers for different genomic regions using primers described in Table S2.

Behavioral Studies

Rotarod

Deterioration of motor function was tested using the accelerating Rotarod in mutant and control mice (n = 4-19/group) each month from ages 1 to 9 months. Mice were subjected to three training trials in order to familiarise them with the procedure 24 hours prior to each recording. Over a 5-minute period, Rotarod speed was gradually increased from 5 to 50 rpm and the time taken to fall recorded. The mice were subject to three trials for each time point, and the average of these trials reported.

Grid Test

The grid test was performed as previously described (Lin et al., 2016). Mice were placed in the center of an elevated wire grid and video recorded by a camera positioned underneath the mesh for 3 minutes. Foot faults were defined as when a hindpaw or forelimb of the mouse fell through the grid while walking. The cumulative time with trapped limbs and the number of grid foot faults was recorded.

QUANTIFICATION AND STATISTICAL ANALYSIS

Statistical analysis was carried out using Prism statistical analysis software. All data are expressed as mean ± SEM unless otherwise indicated. Data were analyzed using a one-way or two-way analysis of variance (ANOVA), followed by Tukey's multiple comparisons test, unpaired two-tailed Student's t test or Mann-Withney test as appropriate. p values are indicated by asterisks as follows: *$p < 0.05$, **$p < 0.01$, ***$p < 0.001$.

Cell Reports, Volume *25*

Supplemental Information

HDAC3 Regulates the Transition to the Homeostatic Myelinating Schwann Cell State

Laura H. Rosenberg, Anne-Laure Cattin, Xavier Fontana, Elizabeth Harford-Wright, Jemima J. Burden, Ian J. White, Jacob G. Smith, Ilaria Napoli, Victor Quereda, Cristina Policarpi, Jamie Freeman, Robin Ketteler, Antonella Riccio, and Alison C. Lloyd

Figure S1

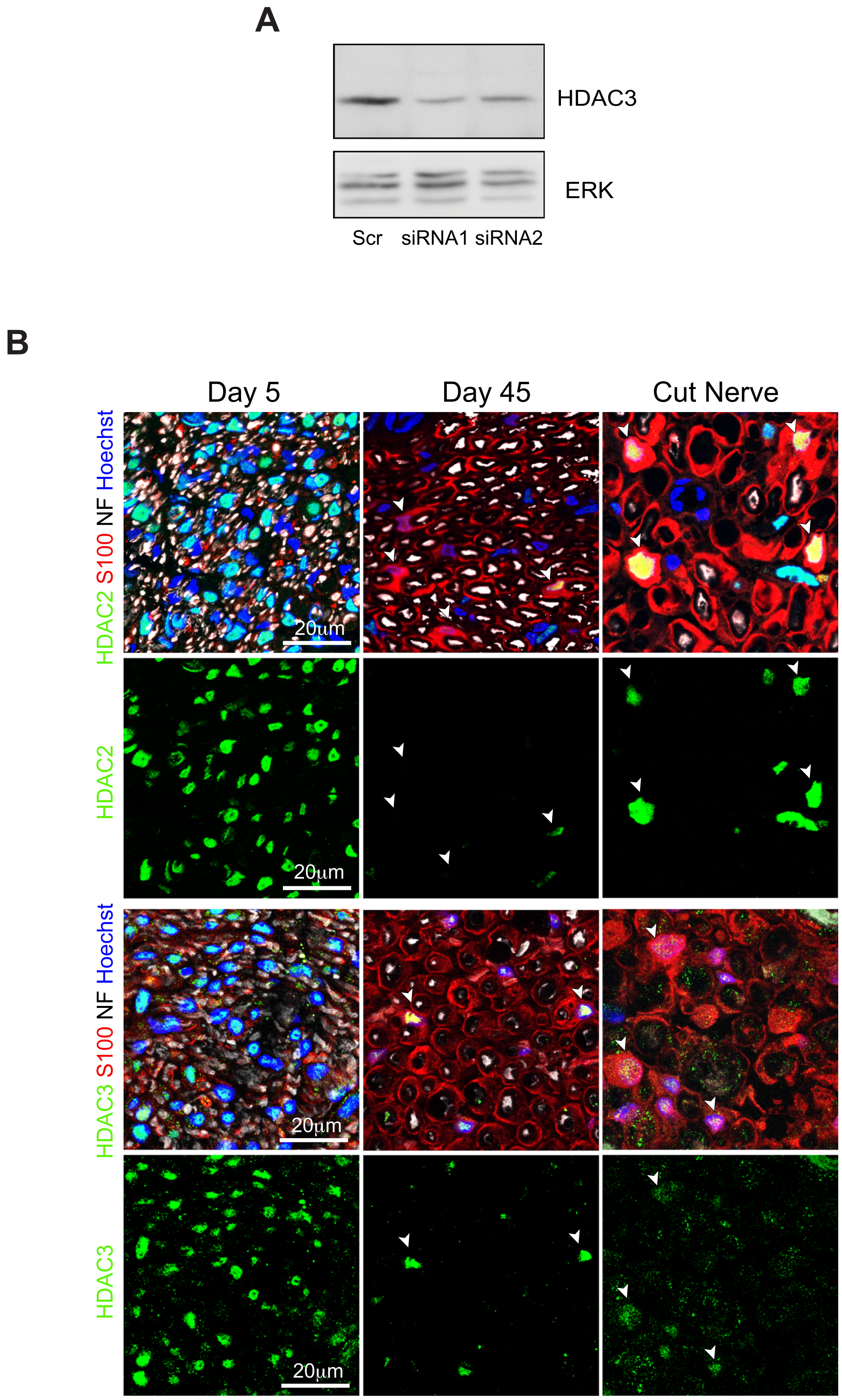
A
HDAC3
ERK
Scr
siRNA1
siRNA2
B
Day 5
Day 45
Cut Nerve
HDAC2 S100 NF Hoechst
HDAC2
HDAC3 S100 NF Hoechst
HDAC3
20µm
20µm
20µm
20µm

Supplementary Figure 1. HDAC3 regulates myelin gene transcription and is expressed in adult myelinating Schwann cells, related to Figure 1. A) Western blot analysis of total protein lysates from siRNA-treated Schwann cells showing the efficiency of HDAC3 knockdown with two independent oligos compared to scrambled control at 48 hr. Total ERK levels were used as a loading control. B) Representative confocal images of transverse sections of rat sciatic nerves at postnatal Day 5, Day 45, and Day 45 animals, 72 hours following nerve transection stained for S100 (red), neurofilament (white) and HDAC2 (top panel) or HDAC3 (lower panel) (green), as indicated. Nuclei were counterstained with Hoechst (blue). Note that similarly to mice (Figure 1D), HDAC2 expression is low in adult mSCs whereas it is reinduced upon injury (arrowheads). Conversely HDAC3 expression is maintained in adult mSCs (arrowheads) whereas it decreases upon injury (arrowheads).

Figure S2

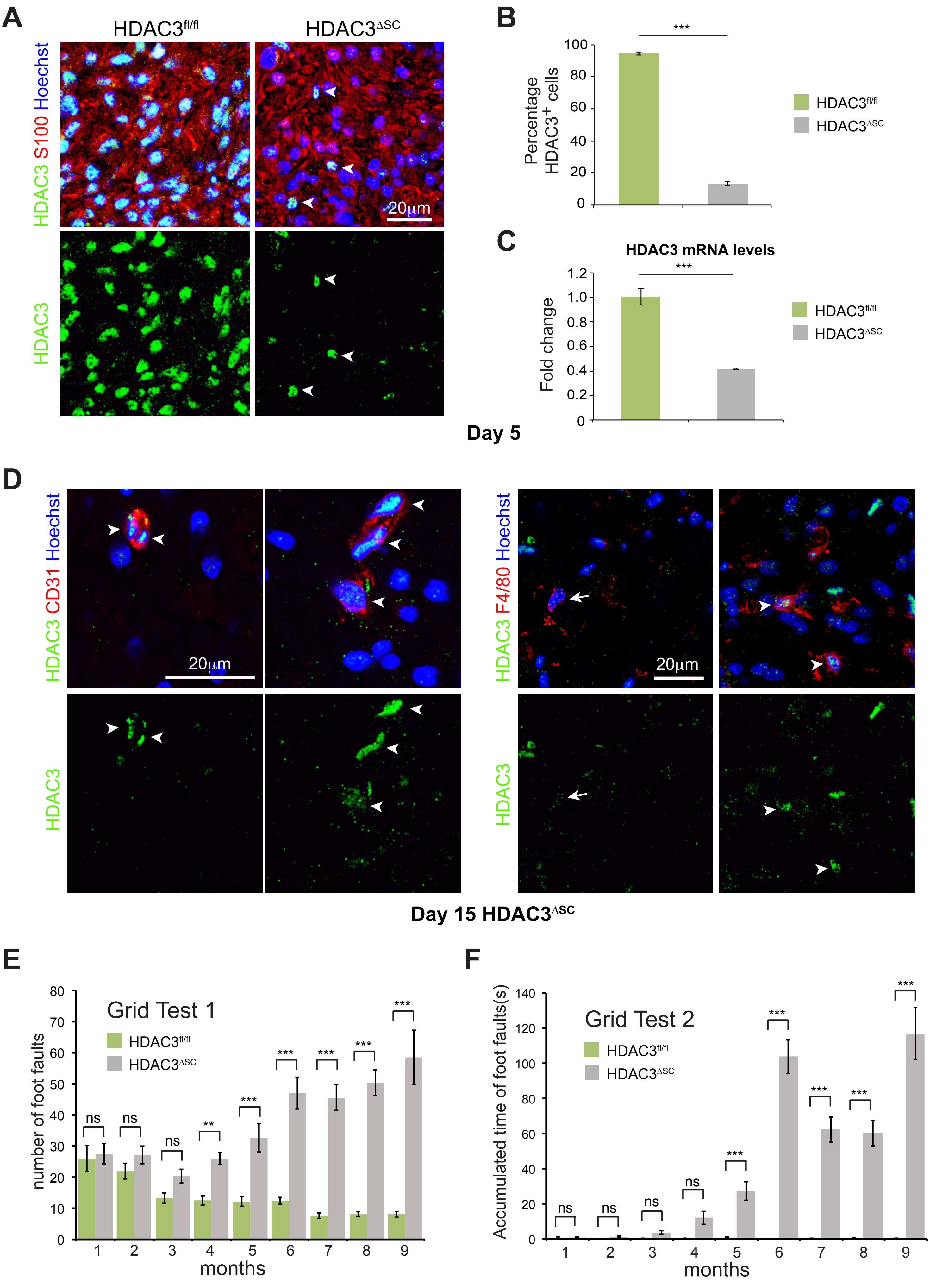
A
HDAC3fl/fl
HDAC3ΔSC
HDAC3 S100 Hoechst
HDAC3
20μm
B
***
Percentage HDAC3+ cells
100
80
60
40
20
0
HDAC3fl/fl
HDAC3ΔSC
C
HDAC3 mRNA levels
***
Fold change
1.2
1.0
0.8
0.6
0.4
0.2
0
HDAC3fl/fl
HDAC3ΔSC
Day 5
D
HDAC3 CD31 Hoechst
HDAC3
20μm
HDAC3 F4/80 Hoechst
HDAC3
20μm
Day 15 HDAC3ΔSC
E
Grid Test 1
HDAC3fl/fl
HDAC3ΔSC
number of foot faults
80
70
60
50
40
30
20
10
0
ns
ns
ns
**
***
***
***
***
***
1
2
3
4
5
6
7
8
9
months
F
Grid Test 2
HDAC3fl/fl
HDAC3ΔSC
Accumulated time of foot faults(s)
140
120
100
80
60
40
20
0
ns
ns
ns
ns
***
***
***
***
***
1
2
3
4
5
6
7
8
9
months

Supplementary Figure 2. Loss of HDAC3 in Schwann cells results in progressive adult neuropathy, related to Figure 2. A) Immunofluorescence of representative transverse sciatic nerve sections from control ($HDAC3^{fl/fl}$) mice or mutant mice ($HDAC3^{\Delta SC}$) at postnatal Day 5 showing efficient loss of nuclear HDAC3 staining (green) in S100-labelled Schwann cells (red). Nuclei were counterstained with Hoechst (blue). Arrowheads point to other cell types that still express HDAC3 in mutant mice. B) Graph shows quantification of (A), which indicates a significant decrease of cells expressing HDAC3 in mutant compared to control mice (n=4, mean±SEM). (C) RT-qPCR analysis of HDAC3 mRNA levels in control ($HDAC3^{fl/fl}$) and mutant mice ($HDAC3^{\Delta SC}$) at postnatal Day 5 (n=3, mean±SEM). (D) Representative confocal images of transverse sections of sciatic nerve from mutant mice ($HDAC3^{\Delta SC}$) at postnatal Day 15 showing HDAC3 staining (green) in endothelial cells labelled with CD31 and indicated with arrowheads (left panel) and in macrophages labelled with F4/80 (right panel) (red). Note only some macrophages are positive for HDAC3 with arrowheads marking HDAC+ macrophages and an arrow indicating a HDAC3- macrophage. Nuclei were counterstained with Hoechst (blue). E) and (F) Grid behavioural test showing (E) average number and (F) accumulated time of foot faults in seconds of control ($HDAC3^{fl/fl}$) and mutant ($HDAC3^{\Delta SC}$) animals from 1-9 months after birth (n=4-19 animals/time point, mean±SEM). $^{**}p<0.01$, $^{***}p<0.001$.

Movie S1, related to Figure 2. Adult HDAC3$^{\Delta SC}$ mice display severe motor disabilities.

Video showing an example of a 9 month-old mutant HDAC3$^{\Delta SC}$ mouse showing symptoms consistent with an advanced neuropathy.

Figure S3

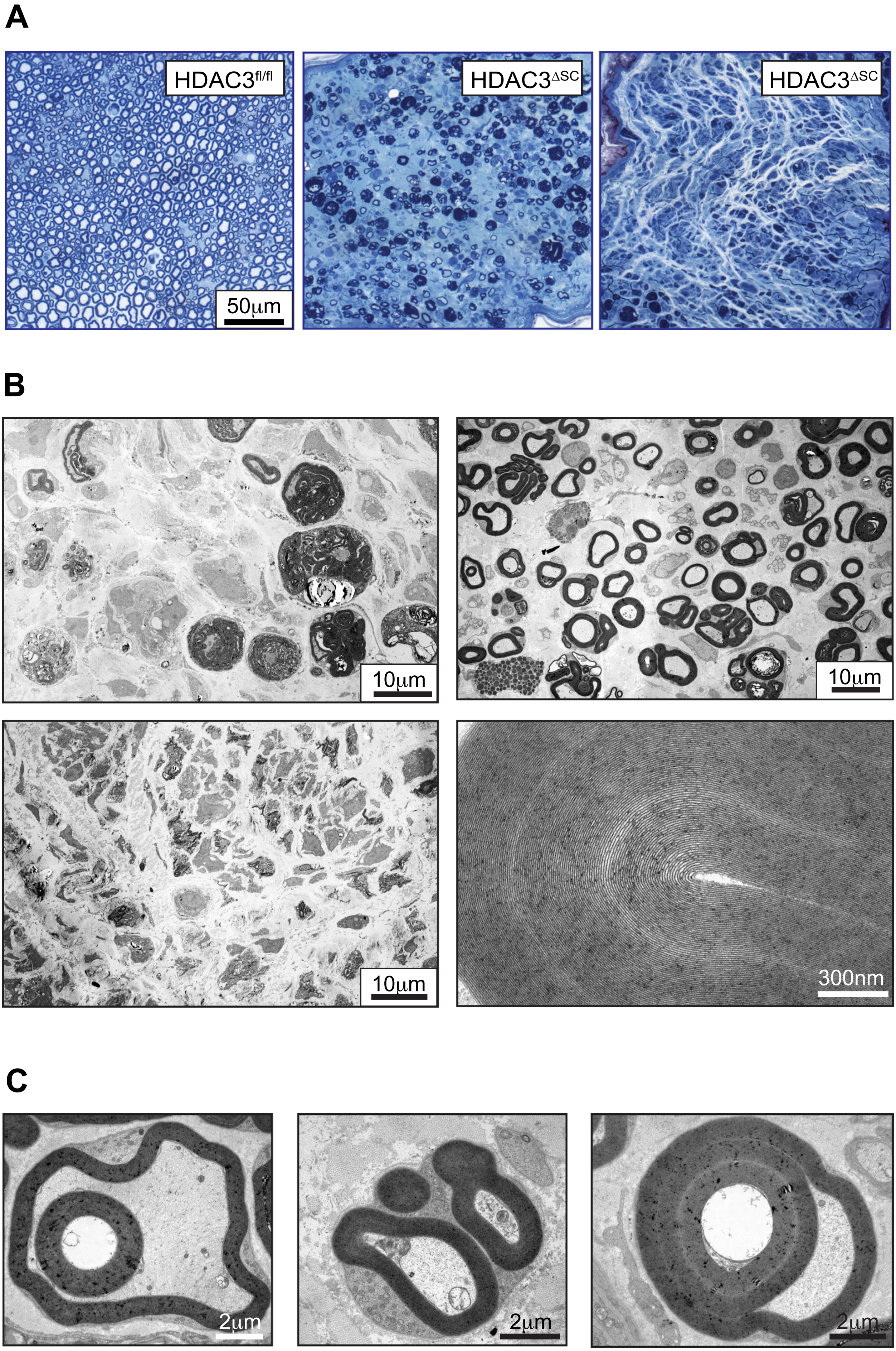
A
HDAC3fl/fl
HDAC3ΔSC
HDAC3ΔSC
50μm
B
10μm
10μm
10μm
300nm
C
2μm
2μm
2μm

Supplementary Figure 3. HDAC3 loss in Schwann cells results in gross myelinating abnormalities, related to Figure 3. (A) Representative images of transverse sections of a sciatic nerve from 9 month-old control $HDAC3^{fl/fl}$ and mutant $HDAC3^{\Delta SC}$ mice stained with toluidine blue, when the mutant animals exhibit severe neuropathies. The left image from a mutant animal shows a decrease in myelinated fibre number and myelination abnormalities, the right image shows fibrotic tissue and predominantly naked axons in more extreme cases. (B) Representative EM images of transverse ultrathin sections showing a collection of gross abnormalities in SC-axonal units in mutant $HDAC3^{\Delta SC}$ mice. Left panels show examples of fibrotic tissue resulting from Schwann cell death and axonal degeneration. The top right panel illustrates abnormalities due to hypermyelination such as tomacula, outfoldings and infoldings as well as inflammatory cell recruitment. The bottom right panel shows normal structure and compaction of myelin in mutant $HDAC3^{\Delta SC}$ mice. (C) High magnification EM images showing myelin infoldings (left and right panel) and outfoldings (middle panel).

Figure S4

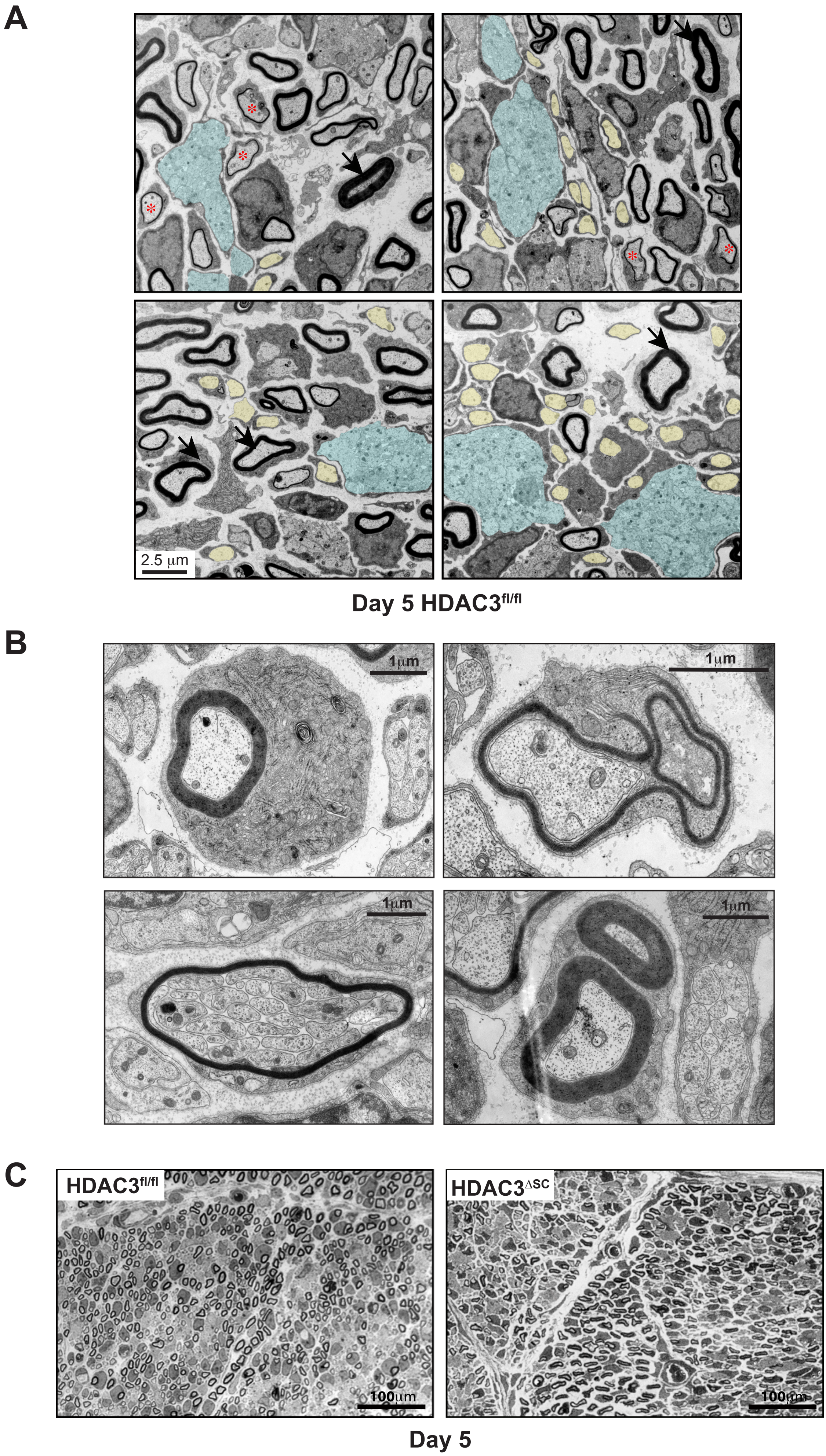
A
2.5 μm
Day 5 HDAC3fl/fl
B
1μm
1μm
1μm
1μm
C
HDAC3fl/fl
HDAC3ΔSC
100μm
100μm
Day 5

Supplementary Figure 4. Myelination initiates normally in Schwann cells lacking HDAC3, related to Figure 4. (A) Representative EM images of transverse ultrathin sections of sciatic nerves from postnatal Day 5 control (HDAC3$^{fl/fl}$) mice. Note that myelination is not a synchronous process: some large and small axons remain unsorted (blue), many Schwann cells have sorted axons and have associated in a 1:1 ratio, but have not yet started the myelination process (yellow), others have just started the myelination process (red *), whereas others have nearly completed myelination (arrows). (B) High magnification EM images of a sciatic nerve from a postnatal Day 5 control HDAC3$^{fl/fl}$ animal showing Schwann cells myelinating normally (top left panel) or abnormally (right top and bottom panels). The bottom left panel shows a Schwann cell myelinating several small diameter axons. C) Representative images of transverse sections of a sciatic nerve from postnatal Day 5 control HDAC3$^{fl/fl}$ and mutant HDAC3$^{\Delta SC}$ animals stained with toluidine blue.

Figure S5

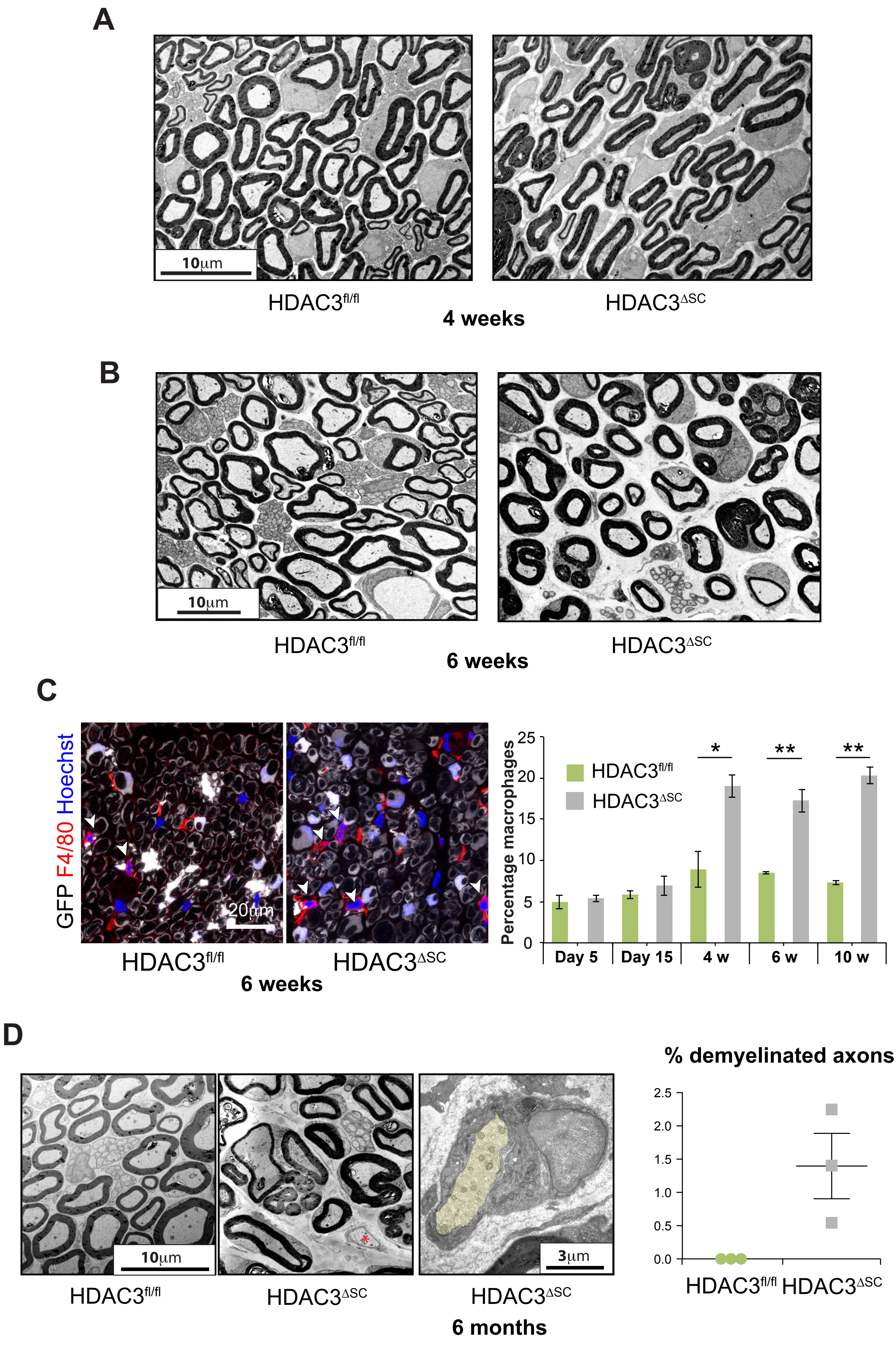

A
10μm
HDAC3fl/fl
HDAC3ΔSC
4 weeks
B
10μm
HDAC3fl/fl
HDAC3ΔSC
6 weeks
C
GFP F4/80 Hoechst
20μm
HDAC3fl/fl
HDAC3ΔSC
6 weeks
Percentage macrophages
25
20
15
10
5
0
HDAC3fl/fl
HDAC3ΔSC
*
**
**
Day 5
Day 15
4 w
6 w
10 w
D
10μm
3μm
HDAC3fl/fl
HDAC3ΔSC
HDAC3ΔSC
6 months
% demyelinated axons
2.5
2.0
1.5
1.0
0.5
0.0
HDAC3fl/fl
HDAC3ΔSC

Supplementary Figure 5. Schwann cells lacking HDAC3 fail to enter the homeostatic state, related to Figure 6. (A) and (B) Representative EM images of transverse ultrathin sections of a sciatic nerve from 4 week (A) and 6 week (B) $HDAC3^{fl/fl}$ and mutant $HDAC3^{\Delta SC}$ animals showing worsening of the phenotype overtime. C) Representative immunofluorescent images of transverse sciatic nerve sections labelled to identify macrophages (F4/80, green) and nuclei (Hoescht, blue) in plp-eGFP (SCs GFP+ in white) control ($HDAC3^{fl/fl}$) and mutant mice ($HDAC3^{\Delta SC}$). Graph shows quantification of macrophages at indicated ages in control ($HDAC3^{fl/fl}$) and mutant mice ($HDAC3^{\Delta SC}$) (n=3 or 4 mean±SEM). D) Representative EM images of transverse ultrathin sections of a sciatic nerve from 6 month-old control ($HDAC3^{fl/fl}$) and mutant mice ($HDAC3^{\Delta SC}$) showing a demyelinated axon in mutant mice ($HDAC3^{\Delta SC}$) (red *) (left panel), a high magnification of a demyelinated axon (yellow) found in a mutant mouse ($HDAC3^{\Delta SC}$) (middle panel) and the quantification of demyelinated axons at that age (n=3 mean±SEM). Note that we did not find any demyelinated axons in the control animals. *$p<0.05$, **$p<0.01$.

Movie S2, related to Figure 5. mSCs lacking HDAC3 develop normally. Movie shows serial EM images of entire mSCs in postnatal Day 15 control (HDAC3$^{fl/fl}$) and mutant (HDAC3$^{\Delta SC}$) mice. Serial 70nm ultrathin longitudinal sections of sciatic nerves were imaged and then aligned. Black arrowheads indicate the mSCs that were used to generate the 3D-reconstructions shown in Figure 5E.

Figure S6

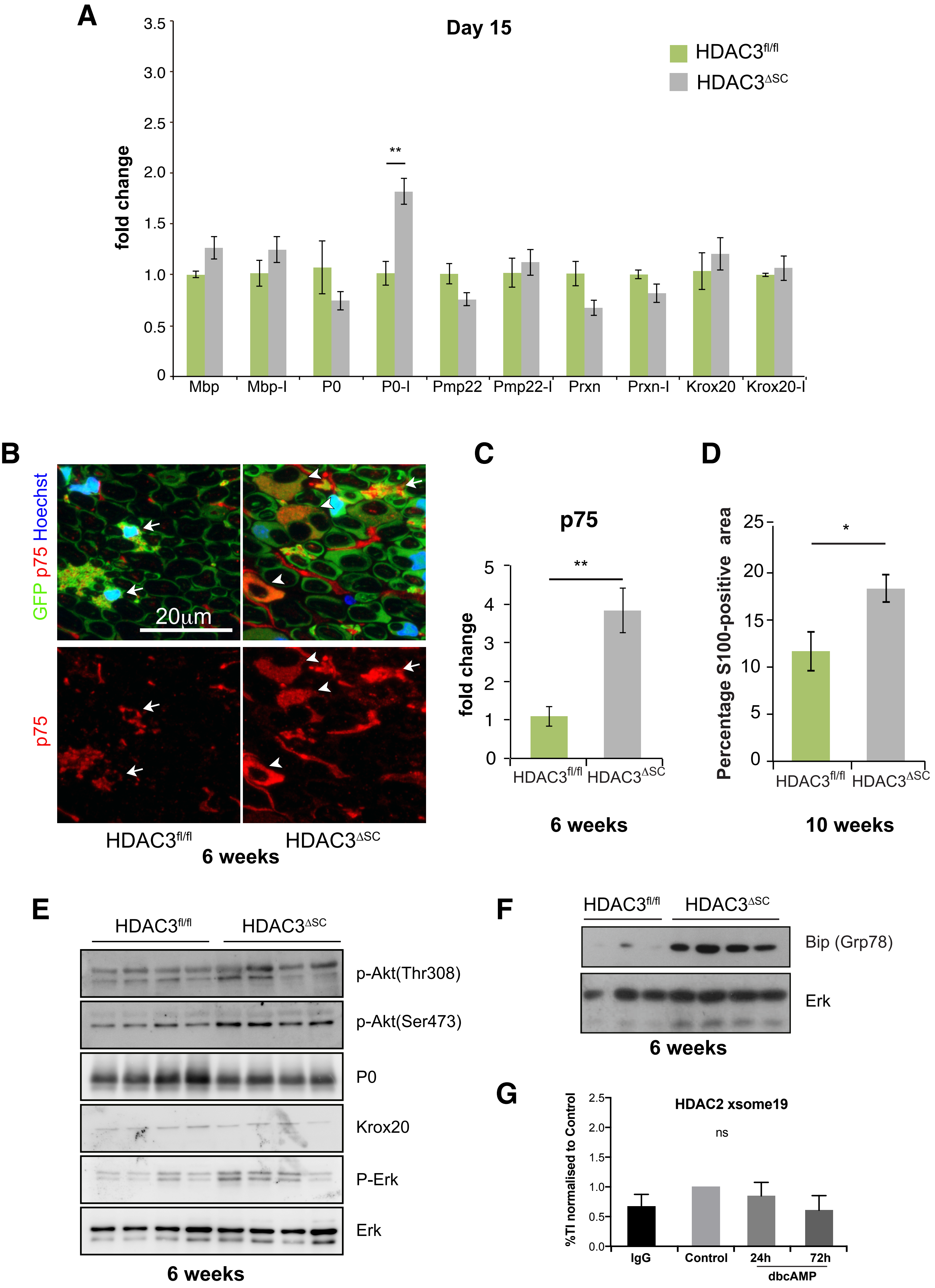
A
Day 15
HDAC3fl/fl
HDAC3ΔSC
fold change
3.5
3.0
2.5
2.0
1.5
1.0
0.5
0
**
Mbp
Mbp-I
P0
P0-I
Pmp22
Pmp22-I
Prxn
Prxn-I
Krox20
Krox20-I
B
GFP p75 Hoechst
p75
20µm
HDAC3fl/fl
HDAC3ΔSC
6 weeks
C
p75
fold change
5
4
3
2
1
0
**
HDAC3fl/fl
HDAC3ΔSC
6 weeks
D
Percentage S100-positive area
25
20
15
10
5
0
*
HDAC3fl/fl
HDAC3ΔSC
10 weeks
E
HDAC3fl/fl
HDAC3ΔSC
p-Akt(Thr308)
p-Akt(Ser473)
P0
Krox20
P-Erk
Erk
6 weeks
F
HDAC3fl/fl
HDAC3ΔSC
Bip (Grp78)
Erk
6 weeks
G
HDAC2 xsome19
%TI normalised to Control
2.5
2.0
1.5
1.0
0.5
0.0
ns
IgG
Control
24h
72h
dbcAMP

Supplementary Figure 6. Myelinating Schwann cells lacking HDAC3 remain in the biogenic state, related to Figure 7. A) RT-qPCR analysis of key myelin genes and the Krox-20 transcription factor in postnatal Day 15 $HDAC3^{fl/fl}$ and mutant $HDAC3^{\Delta SC}$ mice. Graph shows the mRNA or nascent pre-mRNA (-I) levels of mutant sciatic nerves relative to the controls (n = 3 for $HDAC3^{fl/fl}$, n=4 for $HDAC3^{\Delta SC}$, 3 animals were pooled for each sample, mean ± SEM). B). Representative immunofluorescent images of transverse sciatic nerve sections labelled with p75 (red) to identify non-myelinating Schwann cells (arrows) and mSCs that have dedifferentiated (arrowheads) in 6 weeks old plp-eGFP (GFP+ SCs in green) control ($HDAC3^{fl/fl}$) and mutant mice ($HDAC3^{\Delta SC}$). Nuclei were counterstained with Hoechst (blue). C) Graph shows p75 mRNA levels of mutant sciatic nerves relative to the controls at 6 weeks (n = 4 for $HDAC3^{fl/fl}$, n=4 for $HDAC3^{\Delta SC}$, 2 animals were pooled for each sample, mean±SEM) D) Quantification of Figure 7B showing the proportion of S100-positive area in 10 week-old $HDAC3^{fl/fl}$ and $HDAC3^{\Delta SC}$ animals. Note that the cytoplasm of Schwann cells is significantly enlarged in mSCs lacking HDAC3 (n=3 mean±SEM). E) Western blot analysis of total protein lysates from 4 week-old $HDAC3^{fl/fl}$ and mutant $HDAC3^{\Delta SC}$ mice showing no changes between the two genotypes in signalling through the ERK or PI-3kinase pathways. F) Western blot analysis of total protein lysates from 6 week-old $HDAC3^{fl/fl}$ and $HDAC3^{\Delta SC}$ mice showing an upregulation of the molecular chaperone protein BiP/Grp78 in mutant $HDAC3^{\Delta SC}$ mice. G) ChIP analysis of HDAC2 on the negative control region (gene desert on Chromosome 19), in NSΔRafER cells cultured with ± dbcAMP for the indicated times (n = 4, mean±SEM). Note that the % total input of each sample was lower than 0.1% indicating there was no binding on this DNA region. $^*p<0.05$, $^{**}p<0.01$.

Table S1 Primer Sequences for qPCR, related to STAR methods

	Forward 5'-3'	Reverse 5'-3'
Mouse myelin binding protein (mbp) mRNA	gaagctcgtcggactctgag	ggcggtgacagactccaag
Mouse myelin protein zero (P0) mRNA	cggacagggaaatctatggtgc	tggtagcgccaggtaaaagag
Mouse peripheral myelin protein 22 (pmp22) mRNA	catcgcggtgctagtgttg	aaggcggatgtggtacagttc
Mouse periaxin (prxn) mRNA	tcagcggcttcaacgtagc	tagctgccggtgagtcctc
Mouse early growth response protein 2 (Krox20) mRNA	gccaaggccgtagacaaaatc	ccactccgttcatctggtca
Mouse myelin binding protein (mbp) pre-mRNA	caaagcgcaaaagttcccga	cgtgggatacacgagacctg
Mouse myelin protein zero (P0) pre-mRNA	gagattcgggacaatgagggg	aggaggaggcaaccaaacaa
Mouse peripheral myelin protein 22 (pmp22) pre-mRNA	tgttgctgcacttctttgcc	ccttgctcactgtctacccc
Mouse early growth response protein 2 (Krox20) pre-mRNA	atcgccccaaagtgaacagg	cctaggcattctccttgccg
Mouse p75 neurotrophine receptor (NTR) mRNA	acattccgaccgctgatgtt	gacacagaggccctacacag
Mouse Histone deacetylase 3 (hdac3) mRNA	aatgtgcccttacgagatgg	gtagccaccacctcccagta
Mouse beta-2 microglobulin (b2m) mRNA	ttctggtgcttgtctcactga	cagtatgttcggcttcccattc
Rat myelin protein zero (P0) mRNA	ctggtccagtgaatgggtct	catgtgaaagtgccgttgtc
Rat myelin protein zero (P0) pre-mRNA	gaccatatttggcaaggggc	gggtgtcgtctcacgtagc
Rat beta-2 microglobulin (b2m) mRNA	tgaccgtgatctttctggtg	atttgaggtgggtggaactg

Table S2 Primer Sequences for ChIP, related to STAR methods

	Forward 5'-3'	Reverse 5'-3'
P0 enhancer	gtagttatgagcccccagca	tcaccctctccttggtcatc
P0 promoter	tttctgtccctctgcctcac	gtcctgagccagtgaccaa
Xsome19 gene desert	agcctactttcttggcttgc	atgaagctgggctagcagat
